# Genetic and Phenotypic Changes Related to the Development of *mec*-Independent Oxacillin Non-Susceptibility in ST8 *Staphylococcus aureus* Recovered after Antibiotic Therapy in a Patient with Bacteremia

**DOI:** 10.3390/antibiotics13060554

**Published:** 2024-06-13

**Authors:** Sabrina Di Gregorio, Gabriela Weltman, Carolina Fabbri, Silvina Fernández, Soledad Zárate, Jorgelina Smayevsky, Pablo Power, Josefina Campos, Leticia Irene Llarrull, Marta Mollerach

**Affiliations:** 1 Instituto de Investigaciones en Bacteriología y Virología Molecular (IBaViM), Facultad de Farmacia y Bioquímica, Universidad de Buenos Aires, Ciudad Autómoma de Buenos Aires 1113, Argentina; 2 Consejo Nacional de Investigaciones Científicas y Técnicas (CONICET), Ciudad Autónoma de Buenos Aires 1113, Argentina; 3 Instituto de Biología Molecular y Celular de Rosario (IBR, CONICET-UNR), Predio CONICET Rosario, 27 de Febrero 210 bis, Rosario 2000, Argentina; 4 Laboratorio de Bacteriología, Micología y Parasitología, Centro de Educación Médica e Investigaciones Clínicas “Norberto Quirno” (CEMIC), Ciudad Autónoma de Buenos Aires 1431, Argentina; 5 Unidad Operativa Centro Nacional de Genómica y Bioinformática, ANLIS Dr. Carlos G. Malbrán, Ciudad Autónoma de Buenos Aires 1282, Argentina; 6 Área Biofísica, Facultad de Ciencias Bioquímicas y Farmacéuticas, Universidad Nacional de Rosario, Suipacha 570, Rosario 2000, Argentina

**Keywords:** *S. aureus*, MIONSA, WGS, ST8, PBP2

## Abstract

The *mec*-independent oxacillin non-susceptible *S. aureus* (MIONSA) strains represent a great clinical challenge, as they are not easily detected and can lead to treatment failure. However, the responsible molecular mechanisms are still very little understood. Here, we studied four clinical ST8-MSSA-*t024* isolates recovered during the course of antibiotic treatment from a patient suffering successive episodes of bacteremia. The first isolates (SAMS1, SAMS2, and SAMS3) were susceptible to cefoxitin and oxacillin. The last one (SA2) was susceptible to cefoxitin, resistant to oxacillin, lacked *mec* genes, and had reduced susceptibility to teicoplanin. SA2 showed higher β-lactamase activity than SAMS1. However, β-lactamase hyperproduction could not be linked to oxacillin resistance as it was not inhibited by clavulanic acid, and no genetic changes that could account for its hyperproduction were found. Importantly, we hereby report the in vivo acquisition and coexistence of different adaptive mutations in genes associated with peptidoglycan synthesis (*pbp2*, *rodA*, *stp1*, *yjbH*, and *yvqF*/*vraT*), which is possibly related with the development of oxacillin resistance and reduced susceptibility to teicoplanin in SA2. Using three-dimensional models and PBP binding assays, we demonstrated the high contribution of the SA2 PBP2 Ala450Asp mutation to the observed oxacillin resistance phenotype. Our results should be considered as a warning for physicians and microbiologists in the region, as MIONSA detection and treatment represent an important clinical challenge.

## 1. Introduction

*Staphylococcus aureus*, a relevant opportunistic pathogen and a prevalent cause of invasive infections such as bacteremia, has an extraordinary capacity to adapt to different environmental conditions and to acquire antimicrobial resistance.

The emergence and dissemination of Methicillin-Resistant *S. aureus* (MRSA) is of relevant concern worldwide due to its high morbidity and mortality [1]. The molecular mechanism for methicillin resistance involves the acquisition of PBP2a, an alternative penicillin-binding protein encoded by the *mecA* gene (or its homologs *mecC*, *mecB*, *mecD*) [2], conferring resistance to nearly all β-lactam antibiotics (except to ceftaroline and ceftobiprole).

In parallel, strains lacking the *mec* gene but phenotypically resistant to oxacillin (an anti-staphylococcal β-lactam) have been described since 1980 with variable frequency around the globe [3,4,5,6,7,8,9]. The phenotype was initially called “borderline oxacillin-resistant *Staphylococcus aureus*” (BORSA) or “modified PBP *S. aureus*” (MODSA), and more recently has been called “*mec*-independent oxacillin non-susceptible *S. aureus*” (MIONSA) [10].

MIONSA strains are misclassified as methicillin-susceptible *S. aureus* (MSSA) by methods currently applied in clinical laboratories (PCR for *mec* genes or using only cefoxitin disk/MIC as a surrogate marker for methicillin resistance), which can lead to treatment failure if using an anti-staphylococcal β-lactam [10,11].

The hyperproduction of BlaZ (PC1) β-lactamase and mutations in PBPs were the first reported changes associated with MIONSA [4,8,12,13]. Nevertheless, it was later shown that the molecular mechanisms leading to this phenotype can be diverse [9,13,14,15,16], reinforcing the need to further analyze them.

The study of this mechanism is especially important considering that the antibiotic treatment and/or sub-inhibitory concentrations of antibiotics reached during infection may promote the emergence of mutations/chromosomal rearrangements [17] throughout the genome of MSSA strains, which are increasing in prevalence and burden [18,19,20] and may give rise to MIONSA.

**Figure 1:**
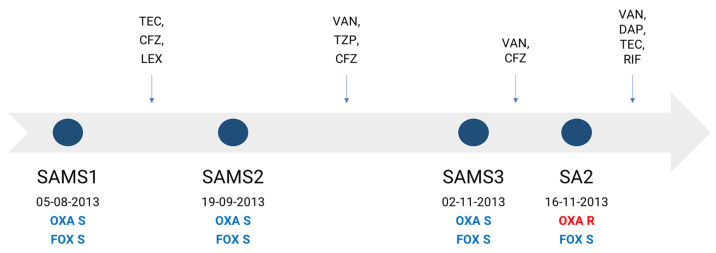
Antibiotic therapy received by the patient and dates of recovery of each *S. aureus* isolate. Antimicrobial therapy is represented above the timeline. OXA, oxacillin; FOX, cefoxitin; TEC, teicoplanin; CFZ, cefazolin; LEX, cephalexin; TZP, piperacillin/tazobactam; VAN, vancomycin; DAP, daptomycin; RIF, rifampin; S, susceptible (blue); R, resistant (red).

In this work, we aimed to characterize the molecular mechanisms associated with the development of oxacillin resistance in a clinical *S. aureus* strain lacking the *mec* gene and recovered during the course of antibiotic treatment from a 61-year-old male suffering successive episodes of bacteremia. The patient was admitted to the hospital for the placement of a stent, after which he developed a superficial vein thrombosis in a peripheral line, with a positive culture for methicillin-susceptible *S. aureus* (MSSA), SAMS1. He received treatment with teicoplanin, cefazolin and cephalexin successively. Forty-five days later, the patient was readmitted due to sepsis without a clear focus. A second MSSA isolate (SAMS2) was recovered from his blood cultures, and he started treatment with vancomycin and piperacillin/tazobactam, later switching to cefazolin until completing 6 weeks of antibiotic treatment. A few days after finishing the treatment, the patient was again readmitted with fever and a third MSSA isolate (SAMS3), and the same antibiotic susceptibility profile as SAMS1 was recovered from 2/2 blood cultures. The patient started treatment with vancomycin and cefazolin. After 14 days, an oxacillin-resistant cefoxitin-susceptible *S. aureus* isolate without any other accompanying resistance (SA2) was recovered from 2/2 blood cultures (Figure 1). The patient received treatment with vancomycin, daptomycin and teicoplanin, which were successively associated with rifampicin; this treatment was completed 6 weeks after the last positive blood culture, evolving favorably.

## 2. Results

### 2.1. Antimicrobial Susceptibility

The β-lactam susceptibility profile received from the hospital was completed by adding different antibiotic susceptibility methods and antimicrobial families (Table 1). We further observed that apart from an increase in oxacillin resistance, SA2 presented increased susceptibility to cefoxitin (higher inhibition zone diameter and a 2-fold decrease in the MIC) and reduced susceptibility to teicoplanin (an 8-fold increase in the MIC and lower prediffusion zone diameter compatible with a probable hVISA phenotype) when compared to SAMS1. In addition, SAMS2 increased the oxacillin MIC compared to SAMS1, but this was within the susceptibility range.

To further evaluate the resistance phenotype, we assayed the susceptibility of SAMS1 and SA2 to other β-lactam antibiotics by disk diffusion, despite the CLSI no longer recommending the use of these disks for *S. aureus* susceptibility testing. SA2 showed a constant decrease in the inhibition zone diameter when compared to SAMS1 for all β-lactam antibiotics tested, except for carbapenems. Moreover, SAMS1 and SA2 displayed a larger inhibition zone diameter with ampicillin–sulbactam than with ampicillin, indicating the presence of a serine-β-lactamase as sulbactam is an inhibitor of this enzyme (Table S1).

### 2.2. Whole Genome Sequence Analysis

In silico genotyping revealed strains belonging to ST8, *spa* type *t*024. They were *lukS/F-PV* negative and related with the previously described CC8e clade [21], when put into a global context with 259 CC8 genomes from 19 countries (Table S2). In particular, strains from this study were more closely related to the CC8-MSSA UP76 strain from Perú (accession GSAMN13979540), and clustered as a monophyletic group, consistent with persistent infection with a MSSA clone (Figure S1).

Whole genome sequences were further analyzed in order to unravel the genetic changes associated with the increase in oxacillin and teicoplanin resistance observed in SA2. The presence of *mecA* and/or other *mec* genes was explored, since this is the most frequent mechanism associated with the oxacillin resistance in *S. aureus*. However, these genes and other parts of the *SCCmec* cassette were not detected.

All strains harbored the *blaZ* gene (PC1, BlaZ type C) associated with penicillin resistance, and a genetic determinant of fosfomycin resistance (*fosB*).

**Figure 2:**
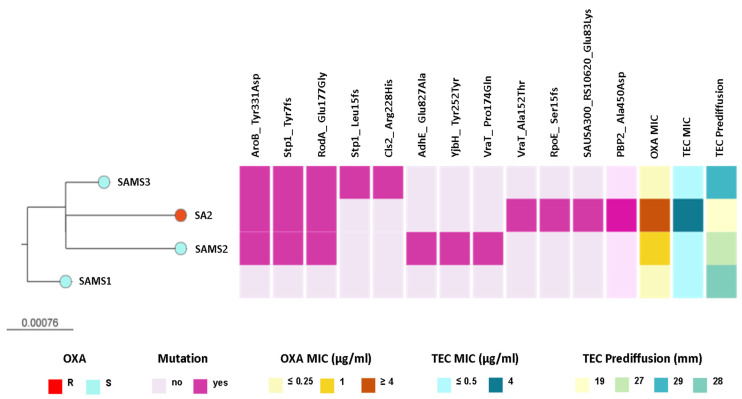
ML phylogenetic tree obtained from core SNP alignment after mapping the reads to the SAMS1 genome assembly. The tree is rooted in the SAMS1 genome. Tree tips are colored by the oxacillin (OXA) resistance phenotype. The scale bar represents the number of single nucleotide polymorphisms (SNPs) per variable site. The distribution of predicted mutations and the profile of susceptibility to OXA and teicoplanin (TEC) are shown as colored blocks, as described in the figure. The figure is available at the following link: https://microreact.org/project/st8-microevolution (accessed on 26 April 2024).

We observed that SAMS2, SAMS3, and SA2 shared genetic changes in AroB (Tyr331Asp), RodA/FtsW (Glu177Gly), and Stp1 (Tyr7fs) when compared with SAMS1. Interestingly, some of these and additional genetic changes that differ between strains occur in genes related with the peptidoglycan metabolism (*rodA*, *stp1*, *vraT*, *yjbH*, *pbp2*, Figure 2 and Table S3).

Interestingly, SAMS2 and SA2, which harbor mutations in both the *vraT* and *stp1* genes (previously linked to the development of reduced glycopeptide susceptibility), also displayed an increased MIC for oxacillin (1 and ≥4 μg/mL, respectively) and reduced susceptibility to teicoplanin. Moreover, the oxacillin resistance and reduced susceptibility to teicoplanin observed in SA2 were associated with additional genetic changes, like the Ala450Asp substitution in PBP2, the major bifunctional peptidoglycan synthase in *S. aureus*, with both transpeptidase and transglycosylase activity (Figure 2 and Table S3).

### 2.3. β-Lactamase Expression

**Figure 3:**
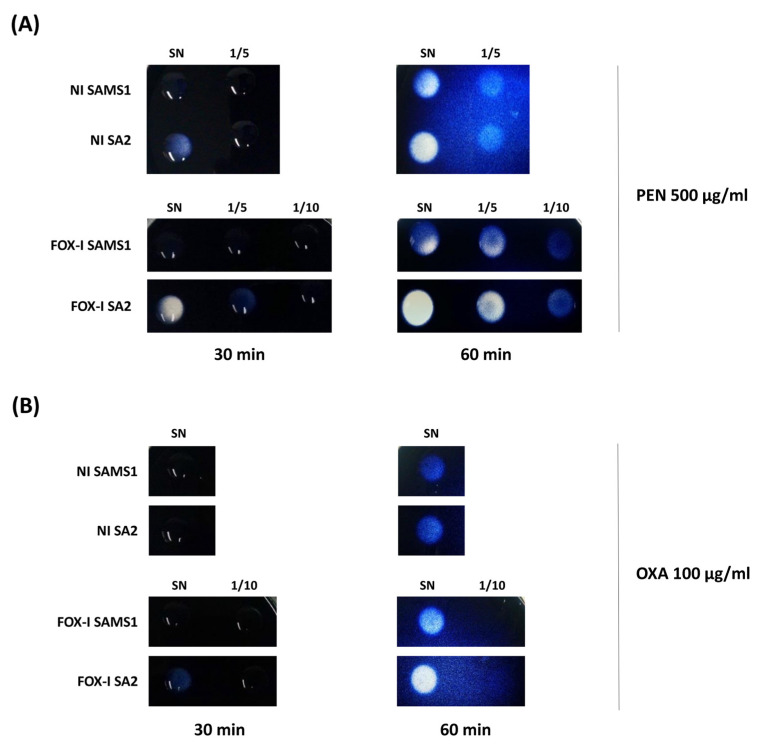
Comparison of β-lactamase activity by the iodometric method. Serial dilutions of the supernatants (1/5, 1/10) of non-induced (NI) and FOX-induced (FOX-I) SAMS1 and SA2 cultures were inoculated to starch agar plates containing either 500 μg/mL of penicillin (PEN) (**A**) or 100 μg/mL of oxacillin (OXA) (**B**). The reaction was followed visually during 60 min. SN = Supernatant.

Considering that the hyperproduction of β-lactamase is a mechanism previously described in MIONSA isolates [4,7], we analyzed *bla* operon sequences and its regulatory genes, which remained unchanged between the four strains. However, semi-quantification of the β-lactamase activity present in the supernatants of SAMS1 and SA2 cultures using starch penicillin agar plates revealed higher penicillinase activity in SA2 (Figure 3A). A slight increase in the β-lactamase (oxacillinase) activity was also observed in starch oxacillin agar plates for the supernatants of SA2 cultures induced with cefoxitin (Figure 3B). The experiment was performed also with starch cefoxitin agar plates, but no cefoxitin hydrolysis was observed after 60 min of incubation. When the same experiment was carried out with 72 h of incubation, the slight hydrolysis was evident with both culture supernatants.

**Figure 4:**
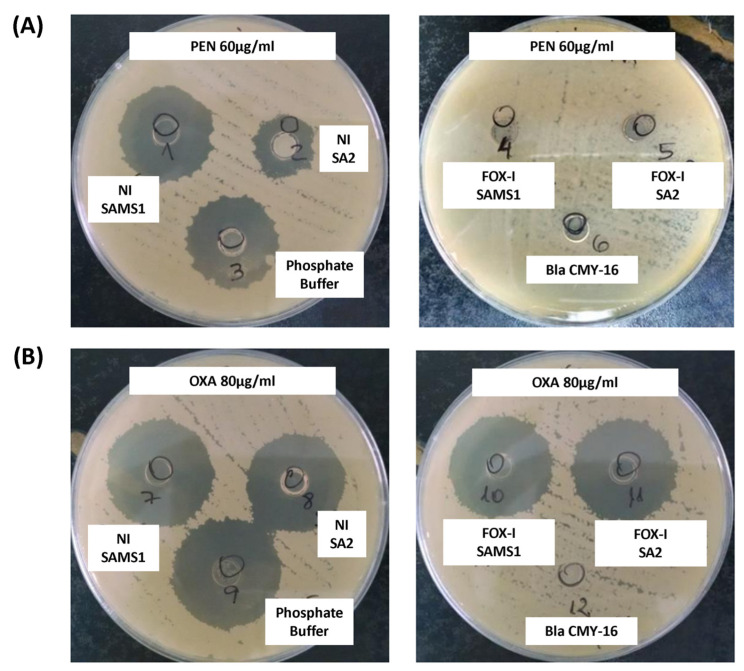
Keserú modified biological assay to detect β-lactamase activity. A mixture of culture supernatant and antibiotic was added to cylinders placed in a plate inoculated with the *B. subtilis* ATCC6633 strain. If a β-lactamase is present in the supernatant, it consumes the antibiotic; any remnant antibiotic can be estimated from the diameter of the inhibition zone in an agar diffusion assay. (**A**) A solution of 60 μg/mL penicillin (PEN) was incubated with the following supernatants: left, non-induced (NI) and right, cefoxitin (FOX)-induced (FOX-I) SAMS1 or SA2 cultures. (**B**) A solution of 80 μg/mL oxacillin (OXA) was incubated with the following supernatants: left, non-induced (NI) and right, FOX-induced (FOX-I) SAMS1 or SA2 cultures. In (**A**,**B**), the controls were as follows: negative control, a solution of 60 μg/mL penicillin (**A**) or 80 μg/mL oxacillin (**B**) incubated with an equal volume of phosphate buffer; β-lactamase positive control, a solution of 60 μg/mL penicillin (**A**) or 80 μg/mL oxacillin (**B**) incubated with purified Bla CMY-16 β-lactamase.

The β-lactamase activity was also assessed in a biological assay. The diameter of the inhibition zone generated after the treatment of PEN with the supernatant of a non-induced SAMS1 culture was comparable to that observed in the absence of β-lactamase activity (control of PEN incubated with buffer; Figure 4A). In contrast, the supernatant of the non-induced SA2 culture showed higher penicillin hydrolyzing activity (lower inhibition zone diameter) than that of the SAMS1 strain (Figure 4A). No remnant penicillin was detected in this assay after treatment with the FOX-induced supernatants of both strains (SAMS1 and SA2), indicating the expression of FOX-induced β-lactamase. In this assay, comparison of the FOX-induced supernatants of the SAMS1 and SA2 cultures did not allow the detection of differences in the level of β-lactamase expression. In addition, this method did not show significant differences in the oxacillin hydrolyzing activity between the supernatants of both non-induced and FOX-induced SAMS1 and SA2 cultures (Figure 4B). Taken together, our results indicate a higher β-lactamase (penicillinase) activity for non-induced SA2 culture.

**Figure 5:**
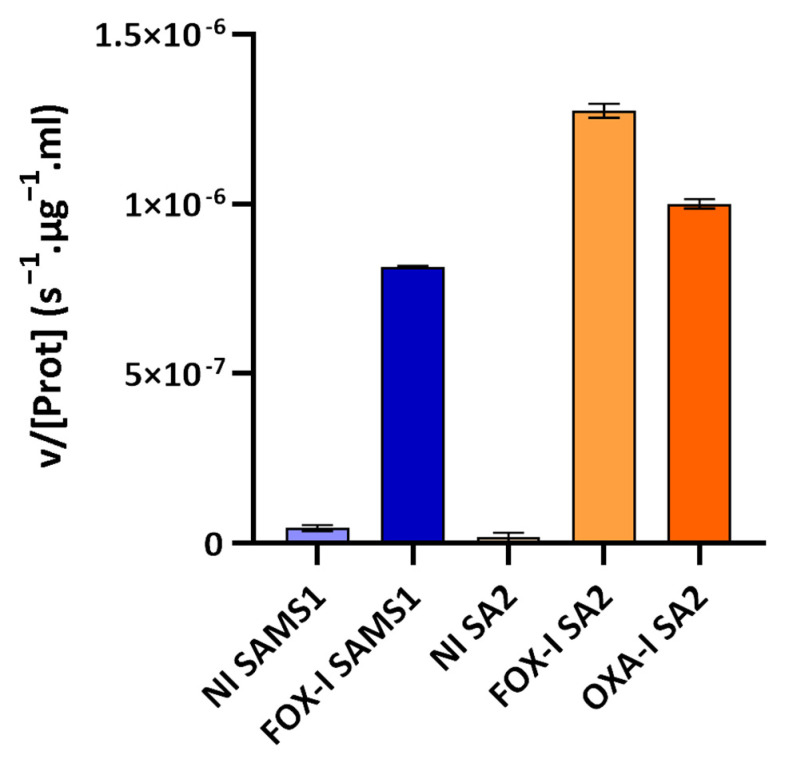
Nitrocefin assay to compare the β-lactamase activity in the supernatants of SAMS1 and SA2 cultures. Hydrolysis of the chromogenic β-lactam antibiotic nitrocefin (88.4 μM) was monitored upon incubation with the supernatants of non-induced (NI), cefoxitin-induced (FOX-I) and oxacillin-induced (OXA-I) cultures. The mean of the initial rate of hydrolysis (expressed as v/[Prot](s^−1^·μg^−1^·mL)) is reported for each culture. At the nitrocefin concentration used in the assay, the initial rate of hydrolysis is a good approximation of the Vmax. Error bars, S.D.

The third approach involved assessing the PC1 β-lactamase activity with nitrocefin as substrate (nitrocefin assay). Nitrocefin is a chromogenic cephalosporin that undergoes a distinctive color change from yellow (λ_max_ = 390 nm at pH 7.0) to red (λ_max_ = 486 nm at pH 7.0) as the amide bond in the β-lactam ring is hydrolyzed by a β-lactamase. Nitrocefin hydrolysis catalyzed by the PC1 β-lactamase, encoded by the *blaZ* gene, was monitored through the increase in absorbance at 485 nm (product absorbance), and the initial rate of product formation was determined. At the nitrocefin concentration used, a saturating one for PC1 β-lactamase, the initial rate measured served as a reliable estimate of Vmax [22]. This rate was directly correlated with the concentration of PC1 β-lactamase in the medium sample. β-lactamase activity was detected in the supernatant of the non-induced and FOX-induced cultures of SAMS1 and SA2 (Figure 5). In the absence of antibiotics, a low-basal β-lactamase activity was registered. Growth in the presence of FOX resulted in an 18-fold increase in the β-lactamase concentration for SAMS1 and a 67-fold increase for SA2 (*p* < 0.0001, ANOVA). For the OXA-resistant SA2 strain, growth in the presence of OXA also resulted in the induction of β-lactamase expression (23-fold increase) (*p* < 0.0001, ANOVA).

Given the higher β-lactamase activity displayed by SA2 using the iodometric method, biological assay and nitrocefin method, we evaluated whether the hyperproduction of β-lactamase could explain the oxacillin resistance, as is the case in some MIONSA strains. The OXA MIC of SA2 remained unchanged in the presence of 4 μg/mL of clavulanic acid (a serine-β-lactamase inhibitor); hence, oxacillin-resistance was ruled out as being significantly influenced by the positive induction of SA2 β-lactamase (Table 1).

### 2.4. Molecular In Silico Modeling of SA2 PBP2

Given the relevance of PBP2 in peptidoglycan synthesis, antimicrobial resistance [23,24], and the proximity of the Ala450Asp substitution to the transpeptidase active site domain of this bifunctional enzyme, we decided to study this change in depth, taking as a reference the sequence of PBP2 (PDB 2OLU).

**Figure 6:**
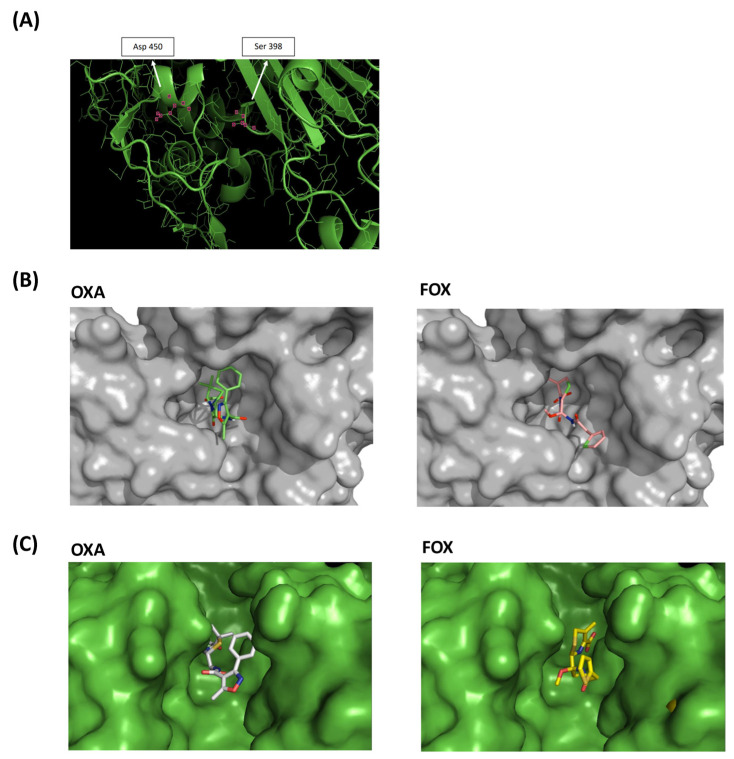
Molecular models of PBP2 Ala450Asp. (**A**) Three-dimensional model of the SA2 PBP2 (Ala450Asp) transpeptidase active site. The positions of the serine active site and the mutated site are marked. (**B**) SA2 PBP2 surface models. (**C**) Surface model of the active site of SA2 PBP2 covalently bound to oxacillin (OXA) and cefoxitin (FOX).

The mutated amino acid in position 450 was found in α-helix 22, between β-chain 12 and loop 25. The mutation was not located in the same pocket where the oxacillin-binding serine active site is. However, using molecular models, we observed that substitution Ala450Asp introduced a charged amino acid into the hydrophobic environment, modified the position of the α-helix 22 and thus impacted the active site cavity (Figure 6A).

The PBP2 surface patterns of SA2 were also obtained in association with oxacillin and cefoxitin molecules, showing the apparent difficulty of accommodating oxacillin in the active site cavity of the transpeptidase, unlike cefoxitin (Figure 6B and Figure S2). Surface models of SA2 PBP2 with covalently bound oxacillin and cefoxitin also evidenced the difficulty of the oxacillin molecule interacting with SA2 PBP2 (Figure 6C).

### 2.5. Effect of the SA2 PBP2 Mutation on β-Lactam Affinity

**Figure 7:**
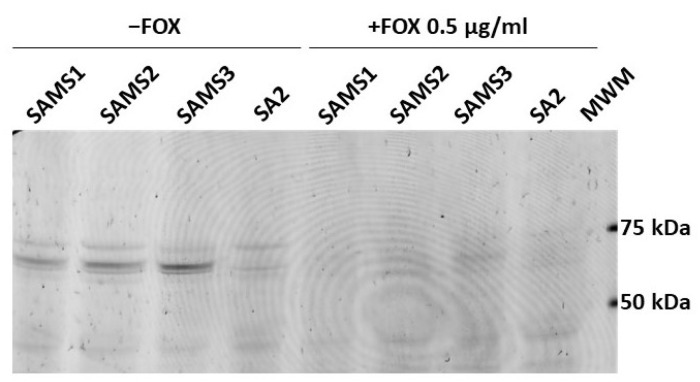
Comparison of the affinity of PBP2 for Bocillin™-FL in *S. aureus* SAMS1, SAMS2, SAMS3, and SA2. The PBPs were labeled with Bocillin™-FL. Lanes 1–4 show the membrane preparations incubated with Bocillin™-FL prior to separation by SDS-PAGE. Lanes 5–8 show the membrane preparations from cultures grown with 0.5 μg/mL cefoxitin (FOX), which were then incubated with Bocillin™-FL. Lane 9. Molecular Weight Markers (MWM); bands at 75, 50 and 25 kDa can be visualized with a 532 nm laser or green LED (FAM filter in Typhoon FLA 7000).

Bocillin™-FL penicillin, a fluorescent derivative of penicillin V with a BODIPY FL moiety, was used to detect the penicillin-binding proteins in the SAMS1, SAMS2, SAMS3, and SA2 membrane preparations (Figure 7). Four Bocillin™-FL-labeled PBP bands were detected in the SAMS1, SAMS2 and SAMS3 membrane protein samples. Based on previous publications, the three stronger bands displayed in the 60–80 kDa were assigned to PBP1 (80 kDa), PBP2 (74 kDa) and PBP3 (70 kDa), with the band corresponding to PBP2 having a higher intensity. The fourth band at ca. 46 kDa could be assigned to PBP4 (46 kDa). Strikingly, the incubation of the *S. aureus* SA2 membrane preparations with the fluorescent penicillin showed the absence of the Bocillin™-FL-labeled PBP2 band at 74 kDa (Figure 7). Preincubation with FOX during culture growth resulted in FOX-bound PBPs in the membrane protein preparations; the binding of FOX to PBPs prevented reaction with Bocillin™-FL and the detection of fluorescent PBPs.

This result suggested that the PBP2 mutation present in SA2 either reduced the affinity for β-lactams so drastically that there was no accumulation of the fluorescent adduct or it interfered with the proper expression of the PBP2 mutant protein in membranes. To rule out the latter possibility, we carried out the in-gel trypsin digestion of bands around 70–75 kDa from the membrane protein samples of cultures grown in the absence of β-lactam antibiotics. The tryptic peptides were separated by reversed-phase LC and characterized by MS/MS. We compared the membranes from SAMS1 and SA2. PBP2 was unequivocally identified in both protein samples, and in the case of the SA2 sample, we were able to detect the PBP2 peptide harboring the Ala450Asp substitution (peptide SHGTVSIYDD450; (Figures S3 and S4). Given that we could confirm that PBP2 Ala450Asp was expressed in SA2, the absence of a Bocillin™-FL-labeled band at ca. 74 kDa indicated that the Ala450Asp substitution strongly affects the affinity of PBP2 for the penicillin Bocillin™-FL. Given the extreme reduction in the affinity of PBP2 Ala450Asp for Bocillin™-FL, it was not possible to assess the affinity of SA2 PBP2 for other β-lactams in competition assays.

## 3. Discussion

This study is the first report of MIONSA in ST8-MSSA-*t*024 developed during the course of antibiotic treatment in vivo in a hospital from Argentina, and its association with reduced teicoplanin susceptibility. Although ST8-MSSA-*t*024 related to the CC8e lineage is not among the most prevalent lineages causing bacteremia in the country [25], our results should serve as a warning for physicians and clinical microbiologists in the region, as the MIONSA phenotype can develop in different MSSA genetic environments [8,13,26] and their detection represents a challenge for clinical laboratories.

Our data also highlight the risk of using a single antibiotic to determine the resistance to an entire antibiotic class, potentially missing unexpected resistant phenotypes. Alternative mechanisms of oxacillin resistance, although infrequent in the clinical laboratory [10], are especially worrisome, given that the resistance to oxacillin would not have been detected by the disk diffusion test (the CLSI recommends using only the cefoxitin disk for MRSA), and in the case of using β-lactam antibiotics, it can lead to therapeutic failure.

In this clinical case of *S. aureus* bacteremia, the mutations associated with oxacillin resistance and a reduced susceptibility to teicoplanin correlated with the prolonged and varied treatment received by the patient, which included β-lactam and glycopeptide antibiotics targeting the bacterial cell wall (Figure 1). An increased rate of adaptive genetic changes was previously found in *S. aureus* causing severe infections [27]. Hence, the genetic changes observed here may have been the result of antibiotic-induced bacterial stress and adaptation, as previously reported by several studies [17,27,28,29,30,31], and may impact the antibiotic resistance phenotype of strains. Alternatively, the oxacillin-resistant *S. aureus* subpopulations harboring genetic changes found here may have been positively selected during the long course of antibiotic treatment [32]

Previous reports have demonstrated that adaptive genetic changes occur at a high frequency in selected MIONSA strains in vitro [10]. Although we did not find changes in the mutational frequency (Table S4), we cannot rule out that the strains analyzed have a hypermutable phenotype, as the state of hypermutability does not always lead to mutations in the *rpoB* gene impacting rifampin resistance (used here as marker of the mutator phenotype).

The presence of the *blaZ* gene in these strains justifies the resistance to penicillins observed [33]. SA2 showed higher β-lactamase activity due to a greater amount of secreted β-lactamase in the growth media, compared to SAMS1. Since no mutations were found in the *bla* operon, the higher β-lactamase expression level seen for SA2 (Figure 3, Figure 4 and Figure 5) may be due to the system crosstalk that occurs during regulation. However, the oxacillinase activity was not significantly higher for SA2, and the oxacillin plus clavulanic acid MIC remained unchanged. Hence, although we confirmed the greater activity of PC1/BlaZ, as in other MIONSA strains [4,12,26], this would not contribute to the oxacillin-resistant phenotype of SA2.

Remarkably, a missense mutation in the *pbp2* gene is among the adaptive changes harbored by SA2. The *pbp2* gene codes for the PBP2 penicillin-binding protein, which is responsible for the transpeptidation and transglycosylation reactions necessary for cell wall synthesis [23,24]. Mutations in PBP genes were previously associated with MIONSA [10,13,26]. Importantly, we here showed that the PBP2 Ala450Asp substitution significantly reduced the affinity of PBP2 to β-lactams, and this seems to highly impact oxacillin resistance.

Although we demonstrated the impact of the PBP2 Ala450Asp substitution, we cannot rule out the effect of other genetic changes on the resistance phenotype (mutations and the mobilization of insertion sequences), as already described [27,34]. We found different copy numbers and/or locations of staphylococcal IS elements (Table S5), and some mutations that might be compensatory. However, we hypothesize that the gradual acquisition and coexistence of selected genomic changes during infection and antibiotic therapy would contribute to the distinctive antibiotic susceptibility profile observed in SA2 (increased oxacillin resistance, increased cefoxitin susceptibility and reduced teicoplanin susceptibility) (Table 1).

Among these are mutations in *rodA*, *stp1*, *yvqF*/*vraT* and *yjbH*, all related with the peptidoglycan metabolism (Figure 2). The strains recovered after antibiotic treatment (SAMS2, SAMS3, SA2) carry mutations in the *stp1* gene, part of the *stk1/stp1* (kinase/phosphatase) system. The mutations in *stp1* and *yjbH* were more likely to arise in severe *S. aureus* infections [10,27]. It has also been reported that the levels of cell wall precursors are significantly altered in *stk1* and/or *stp1* mutants, which are able to modify the structure of the peptidoglycan and the susceptibility to antibiotics [35,36,37,38]. Additionally, *yjbH,* which has previously been linked with PBP4 hyperproduction, oxacillin and vancomycin resistance [13,16,38,39], was mutated in SAMS2. Moreover, the two strains with a higher oxacillin MIC and lower susceptibility to teicoplanin (SAMS2 and SA2) carried mutations in *yvqF/vraT*, which is part of the *vraT-vraS-vraR* operon that controls the expression of genes related to cell wall synthesis (Figure 2). Mutations in this system have been described in association with a decreased susceptibility to vancomycin (hVISA/VISA phenotype) [36,40,41,42], and mutations in *vraT* are among the three most frequent mutations found in selected MIONSA strains in vitro [10].

As far as we know, there are not many studies analyzing the susceptibility to both β-lactam and glycopeptides in MIONSA strains. Nonetheless, the MIONSA and hVISA/VISA phenotypes possess several points in common [10]: they can develop during the course of antibiotic treatment in severe infections; they are associated with antibiotic treatment failure; there is not a unique genetic marker responsible for the phenotype; and genetic changes usually emerge in genetic systems/loci involved in cell wall metabolism. However, the crosstalk between all these genetic systems/loci and their impact on the MIONSA/hVISA/VISA phenotypes are not clearly understood yet [43], highlighting the need for future complementation studies. This is especially important considering that both β-lactams and glycopeptides are used to treat severe *S. aureus* infections, such as the one described in this study.

## 4. Materials and Methods

### 4.1. Bacterial Isolates

Clinical *S. aureus* strains were recovered from blood cultures during the course of antibiotic treatment from a 61-year-old male patient suffering successive episodes of bacteremia. The first recovered isolates (SAMS1, SAMS2, and SAMS3) were susceptible to cefoxitin (FOX) and oxacillin (OXA), while the last one (SA2) was susceptible to FOX and resistant to OXA (Figure 1). Positive blood cultures were subsequently plated in Blood Agar plates (Britania, Buenos Aires, Argentina) and the colony morphology was inspected to verify pure culture. One colony was recovered for each strain. The species identification of isolates was confirmed by conventional biochemical tests and they were stored in BHI (Britania, Buenos Aires, Argentina) + 20% glycerol (Merck, Darmstadt, Germany) at −20 °C for further analysis.

### 4.2. Antimicrobial Susceptibility Tests

Antimicrobial susceptibility was determined using the VITEK 2C automated equipment (Biomerieux, Marcy L’Etoile, France) with the AST-P577 card, following the manufacturer’s instructions, and interpreted according to the CLSI recommendations [44].

Additionally, susceptibility to cefoxitin, oxacillin and tigecycline was evaluated using the disk diffusion method and the MICs for oxacillin (Sigma Aldrich, St. Louis, MO, USA), cefoxitin (Sigma Aldrich, St. Louis, MO, USA), and an oxacillin–clavulanic acid (Sigma Aldrich, St. Louis, MO, USA) combination at 4 μg/mL were determined using the microdilution method. CLSI recommendations and breakpoints were used in all cases [44]. The vancomycin and teicoplanin susceptibility was also studied by the prediffusion method, as previously described [45].

### 4.3. Whole Genome Sequencing and Analysis

Genomic DNA was extracted from overnight BHI (Britania, Buenos Aires, Argentina) cultures using the Wizard^®^ Genomic DNA Purification Kit (Promega, Madison, MI, USA) according to the manufacturer’s instructions, with the addition of lysostaphin (Sigma Aldrich, St. Louis, MO, USA) (0.03 μg/μL) in the lysis step with an incubation time of at least half an hour at 37 °C.

Shotgun gDNA libraries were prepared and whole genome sequencing (WGS) was performed using the Illumina MiSeq platform (paired end, 250 bp) using V2 chemistry. Reads were quality assessed with FASTQC [46] and Kraken2 v2.1.1 [47], and de novo assembled using Unicycler (v0.5.0) [48], excluding contigs of less than 500 bp. After calculating the assembly metrics with Quast v5.2.0 [49], the contigs were annotated with Prokka (1.14.6) [50] and a genus-specific database from RefSeq [51]. In addition, the reads were reference mapped, and variants were named and annotated with snippy v4.6.0 [52] using the assembled genome of the first clinical isolate, SAMS1, as a reference. Alternatively, *S. aureus* USA300_FPR3757 (CC8, Genebank Accession number GCF_000013465.1) was used as a reference sequence. Snippy core SNP alignment was used to build a Maximum Likelihood phylogenetic tree with IQ-Tree v2.1.2 [53], using ModelFinder to determine the best-fit model [54]. Branch support was estimated with the SH-aLRT test and ultrafast bootstrap (1000 replicates each) [55]. All variants (SNPs, INDELs) were manually inspected and visualized with Artemis [56].

The detection of antimicrobial resistance determinants was carried out with ARIBA v2.12.1 [57] and the relevant databases: NCBI [58], Resfinder [59] and CARD [60] (all databases accessed 19 April 2020). Insertion sequences were searched with ISmapper v2.0 [61].

The spa types and MLST types were derived from assemblies using the free online resource spatyper (https://spatyper.fortinbras.us/ accessed on 26 April 2024) and Pathogen Watch (https://pathogen.watch/, accessed on 26 April 2024).

Pathogen Watch was also used to contextualize the genome assemblies with Argentinean and global public genomes from the same clonal complex (CC8) (https://pathogen.watch/collection/s4pbk4850a1j-st8-cemic-update-23-06-2023, accessed on 26 April 2024).

### 4.4. Beta-Lactamase Activity Assays

Three different approaches were used to analyze the level of β-lactamase activity in the *S. aureus* culture media:

The exponential phase cultures of each *S. aureus* strain (OD600 nm = 1) grown with and without FOX induction (0.5 μg/mL) were centrifuged for 10 min at 7000× *g*, and the supernatant was then filtered (0.2 μm filter) to remove any remnant cells, and used to evaluate the β-lactamase activity by alternative methods (biological assay, the iodometric method and nitrocefin assay). Total protein was quantified using the BCA protein assay kit (Pierce, Thermo Fisher Scientific, Rockford, IL, USA). Aliquots containing 50 μg of total proteins were used in the biological and iodometric assays.

#### 4.4.1. Biological Method

The biological method described by Keserú et al. [12] was modified to semi-quantify the production of β-lactamases. A 50 μL aliquot of each filtered culture supernatant (OD600 = 1) (50 μg of total proteins) was mixed with 50 μL of OXA (80 μg/mL) or PENI (60 μg/mL). After a 30 min incubation, the mixture was added to sterile metal cylinders placed on the surface of MHA plates inoculated with a 0.5 Mc Farland suspension of the *B. subtilis* ATCC6633 strain. The residual OXA or PEN activity was evaluated by measuring the inhibition zone diameter after 20–24 h of incubation at 37 °C. Controls were performed by incubating each of the OXA or PENI solutions with 50 μL of 0.05 M phosphate buffer (antibiotic activity control) or with a purified extract of CMY-16 β-lactamase (antibiotic hydrolysis control) for 30 min prior to the inoculation of the plates. In addition, the plates were inoculated directly with each filtered culture supernatant (control of absence of inhibitory activity).

#### 4.4.2. Iodometric Method

For the iodometric method, starch agar plates (0.25 g of starch (Sigma Aldrich, St. Louis, MO, USA), 0.50 g of Agar-Agar (Britania, Buenos Aires, Argentina), 50 mL of 50 mM phosphate buffer pH: 7.5, and 1 mL of a potassium iodide–iodine solution) were prepared with OXA (100 μg/mL), FOX (100 μg/mL) or PEN (500 μg/mL), all from Sigma Aldrich, St. Louis, MO, USA. The plates were then inoculated with 50 μL of undiluted supernatant (45 mg total proteins) and serial dilutions of supernatants, and incubated at 37 °C for 60 min. The presence of β-lactamases in the culture supernatant causes the discoloration of the agar as a consequence of the acidification of the medium upon β-lactam hydrolysis [62]. The progress of the reaction was monitored at 30 and 60 min, and pictures were taken at each time point.

#### 4.4.3. Nitrocefin Assay

A culture of the corresponding *S. aureus* strain was initiated by a 1/100 dilution of a saturated culture (grown overnight at 37 °C, 220 rpm). The cells were grown in LB with or without 0.5 μg/mL of FOX or 0.5 μg/mL of OXA at 37 °C, 220 rpm, until OD600 nm = 1. The supernatant medium was separated from the cells by centrifugation for 10 min at 21,000× *g* and 4 °C. A 500 μL portion of the corresponding supernatant was first equilibrated to room temperature, and the reaction was started by the addition of 9 μL of a 5 mM nitrocefin (Sigma Aldrich, St. Louis, MO, USA) stock solution in dimethyl sulfoxide (DMSO) (final nitrocefin concentration was 88.4 μM). Nitrocefin hydrolysis was monitored at 500 nm in a Jasco V-530 spectrophotometer, for 300 s at room temperature, and the initial rate calculated. At this nitrocefin concentration, the initial rate is a good estimation of the Vmax [22], which is directly proportional to the concentration of PC1 β-lactamase in the medium sample. Total protein was quantified using the BCA protein assay kit (Pierce, Thermo Fisher Scientific, Rockford, IL, USA), after the TCA precipitation of total proteins from the culture supernatant, two washes with cold acetone and resuspension in 2% SDS.

### 4.5. PBP2 In Silico Modeling

The amino acid sequences of PBP2 from SAMS1 and SA2 were used to build a multi-alignment using ClustalW2 (http://www.ebi.ac.uk/Tools/msa/clustalw2/ accessed on 26 April 2024) and Espript 3.0 (http://espript.ibcp.fr/ESPript/ESPript/ accessed on 26 April 2024). The in silico modeling of these proteins was performed using the Swiss-Model server (http://swissmodel.expasy.org/ accessed on 26 April 2024) using the X-ray structure of *S. aureus* PBP2 (PDB 2OLU) as a template. The spatial coordinates of oxacillin (OXA) and cefoxitin (FOX) were retrieved from the ZINC database (https://zinc15.docking.org/ accessed on 26 April 2024), and the structures were minimized in Avogadro v1 [63,64]. The covalently linked structures of the PBP variants with either OXA and FOX were energy minimized with Yasara [65], using a standard protocol consisting of a steepest descent minimization followed by the simulated annealing of the ligand and protein side chains, with the following simulation parameters used: YASARA2 force field, cutoff distance of 6 Å, periodic boundary conditions and water-filled simulation cell. All models were visualized with PyMOL v2.5.2 [66].

### 4.6. Membrane Purification and PBP Analysis

#### 4.6.1. Bocillin™-FL Assay

For each strain, 100 mL of TSB medium (Britania, Buenos Aires, Argentina) in a 500 mL Erlenmeyer flask was inoculated with 100 μL of a stationary phase culture. The cultures were grown at 37 °C, 220 rpm, until A600 reached 0.8 AU. At this point, the cultures were divided into 40 mL aliquots in sterile 250 mL glass bottles. One 40 mL culture was grown at 37 °C, 220 rpm, in the absence of β-lactam antibiotic and the other aliquots were supplemented with 0.5 μg/mL of cefoxitin. After 1 h of growth, when all cultures reached an A600 of 2.5–3, 35 mL aliquots of each culture were centrifuged for 10 min at 7000× *g* and 4 °C, and the cell pellets were stored at −20 °C.

For the detection of PBPs in membrane extracts, the cell pellets were thawed and resuspended in Lysis Buffer 1 (100 mM of sodium phosphate, pH 7.5, 50 mM of NaHCO_3_, 0.5 mM of phenylmethylsulfonyl fluoride (PMSF), 20 mM of MgCl_2_, 15 μg/mL DNase I, 10 μg/mL RNase A, 50 μg/mL lysostaphin) and incubated for 30 min at 37 °C, 220 rpm. The extract was then sonicated in an ice-water bath using a High Intensity Ultrasonic Processor 600 watt—Model 602 (Cole Parmer, Vernon Hills, IL, USA), with the probe tuned to vibrate at 20 kHz ± 50 Hz. Samples were subjected to 6 cycles of 10 s sonication at 30% amplitude with a 30 s break in between. The extracts were ultracentrifuged for 45 min at 150,000× *g* and 4 °C to separate the soluble proteins from the membrane fraction. The total membrane protein was quantified using the BCA protein assay kit (Pierce, Thermo Fisher Scientific, Rockford, IL, USA). Aliquots containing 200 μg of total membrane proteins were incubated with 100 μM of Bocillin™-FL (Life Technologies, Thermo Fisher Scientific, Rockford, IL, USA) at 37 °C for 15 min, and the reaction was stopped by the addition of sample buffer (5% *v*/*v* glycerol, 2% *w*/*v* SDS, 0.1 % *w*/*v* β-mercaptoethanol and 0.1 mg/mL bromophenol blue) and heating for 5 min at 95 °C. Aliquots containing 60 μg of total protein and Molecular Weight Marker: Precision Plus Protein™ WesternC™ Blotting Standards (Bio-Rad Laboratories, Inc., Catalog #1610376, Hercules, CA, USA) were loaded in 12% Tris-glycine SDS-polyacrylamide gels. The fluorescent marks corresponding to the Bocillin™-FL-labeled proteins were detected using a Typhoon™ FLA 7000 laser scanner with the FAM filter at 900 V. After registering the fluorescent image, the gel was stained with Coomassie Brilliant Blue to visualize the total proteins.

#### 4.6.2. In-Gel Trypsin Digestions and LC/MS/MS Analysis

The membrane protein samples of cultures grown in the absence of β-lactam antibiotic, as described in the ‘Bocillin™-FL Assay’ section, were separated in 12% SDS-polyacrylamide gels. Bands around 70 kDa were excised with a clean razor blade, placed into individual tubes, and digested in gel, as described by Link and LaBaer [67]. Mass spectrometric analysis of the peptides was carried out at the Mass Spectrometry Unit of the Institute of Molecular and Cellular Biology of Rosario (UEM-IBR): 4 μL of tryptic peptides from each sample were seeded on a Q-Exactive HF mass spectrometer in positive mode. Peptide separation was performed on an Ultimate3000 nanoHPLC equipped with a 15 cm C18 nano column (ES801, Thermo Fisher Scientific, Rockford, IL, USA). The elution gradient consisted of a mixture of solvents (water, “solvent A” and acetonitrile, “solvent B”, both containing 0.1% formic acid). The data obtained were analyzed in Proteome Discoverer 2.4 using the database corresponding to the *S. aureus* Mu50 strain downloaded from Uniprot, and the sequences of *S. aureus* PBP2 WT and Ala450Asp and the standard search parameters for a Q-Exactive Orbitrap instrument. Up to 2 trypsin missed cleavages were tolerated. In all cases, cysteine carbamidomethylation (fixed mod.) and methionine oxidation (variable mod.) were incorporated as modifications.

### 4.7. Mutation Frequency

The mutation frequency was explored for SAMS1 and SA2 in comparison with *S. aureus* ATCC 25923. Briefly, bacteria were grown in TSB (Britania, Buenos Aires, Argentina) until they reached an OD 620 nm = 0.5–0.7 and plated in TSA (Britania, Buenos Aires, Argentina) with or without rifampin (Sigma Aldrich, St. Louis, MO, USA) (100 μg/mL) for viable count. The experiment was performed by triplicate and the mutation frequency was determined and expressed as the media of M/V, where M was the number of rifampin-resistant mutants and V was the number of viable cells in the culture [68].

### 4.8. Statistical Analysis

Numerical values were compared using the two-way analysis of variance (ANOVA). Multiple comparisons were performed with the Tukey post-test. *p* values of <0.05 were considered statistically significant.

## 5. Conclusions

In this study, we analyzed in depth the microevolution and emergence of oxacillin resistance and the reduced susceptibility to teicoplanin in a subset of ST8 *S. aureus* clinical strains lacking the *mec* gene, recovered from blood cultures during an extended period of antibiotic therapy. To the best of our knowledge, this is the first case of MIONSA reported in our country.

The existence of a single mutation in PBP2 near the transpeptidase active site of PBP2 in SA2 could explain this unusual resistance phenotype (MIONSA). However, the possible coexistence of other molecular mechanisms that contribute to resistance to this antibiotic should not be disregarded, as this work reports the acquisition and coexistence of several genomic changes during antibiotic treatment, some associated with modifications in peptidoglycan synthesis.

Our results should be considered as a warning for physicians and microbiologists in the region, as MIONSA detection and treatment represent an important clinical challenge.

## Figures and Tables

**Table 1:** Antimicrobial susceptibility profile.

Antibiotic	Antibiotic Susceptibility Test	SAMS1	SAMS2	SAMS3	SA2
Cefoxitin	Vitek2 test	neg	neg	neg	neg
	MIC (μg/mL) *	4	ND	ND	2
	Disk diffusion (mm)	24	ND	ND	35
Oxacillin	MIC (μg/mL)	≤0.25	1	≤0.25	≥4
	MIC (μg/mL) *	0.5	ND	ND	16
	Disk diffusion (mm)	15	ND	ND	6
Oxacillin—clavulanic acid	MIC (μg/mL) *	0.25	ND	ND	16
Teicoplanin	MIC (μg/mL)	≤0.5	≤0.5	≤0.5	4
	Prediffusion (mm)	28	27	29	19
Vancomycin	MIC (μg/mL)	1	1	≤0.5	1
	Prediffusion (mm)	26	27	28	25
Gentamicin	MIC (μg/mL)	≤0.5	≤0.5	≤0.5	≤0.5
Ciprofloxacin	MIC (μg/mL)	≤0.5	≤0.5	≤0.5	≤0.5
Levofloxacin	MIC (μg/mL)	≤0.12	≤0.12	≤0.12	≤0.12
Moxifloxacin	MIC (μg/mL)	≤0.25	≤0.25	≤0.25	≤0.25
Erythromycin	MIC (μg/mL)	≤0.25	≤0.25	≤0.25	≤0.25
Clindamycin	MIC (μg/mL)	≤0.25	≤0.25	≤0.25	≤0.25
iMLSb phenotype	Vitek2 test	neg	neg	neg	neg
Quinupristin	MIC (μg/mL)	≤0.25	≤0.25	≤0.25	≤0.25
Linezolid	MIC (μg/mL)	2	2	2	1
Minocycline	MIC (μg/mL)	≤0.5	≤0.5	≤0.5	≤0.5
Tetracycline	MIC (μg/mL)	≤1	≤1	≤1	≤1
Nitrofurantoin	MIC (μg/mL)	≤16	≤16	≤16	≤16
Rifampin	MIC (μg/mL)	≤0.5	≤0.5	≤0.5	≤0.5
TMP	MIC (μg/mL)	≤10	≤10	≤10	≤10
Tigecycline	Disk diffusion (mm)	24	24	25	28

* MIC was determined by the microdilution method, otherwise it was determined by the Vitek2 system. ND = Not Determined. neg = Negative. iMLSb = Inducible Macrolide Lincosamide and Streptogramin B resistance. TMP = Trimethoprim Sulfamethoxazole.

## Data Availability

Genomic reads and assemblies can be found in the National Center for Biotechnology Information (NCBI) genome database in the Sequence Read Archive (SRA) under the BioProject PRJNA1099225 with the following biosample accession numbers: SAMN40932157 (SAMS1), SAMN40932186 (SAMS2), SAMN40932247 (SAMS3), SAMN40932248 (SA2).

## Supplementary Materials

The following supporting information can be downloaded at: https://www.mdpi.com/article/10.3390/antibiotics13060554/s1, Figure S1: CC8 Global Contest; Figure S2: 2D view showing the main interactions involved in the complex between SA2-PBP2 and cefoxitin (FOX) or oxacillin (OXA); Figure S3: LC/MS/MS analysis of PBP2 sequences; Figure S4: Annotated MS/MS spectra of the SHGTVSIYDD450 PBP2 peptide identified in the 70–75 kDa region of membrane protein extract of *S. aureus* SA2; Figure S5: Comparison of the affinity of PBP2 for Bocillin™-FL in *S. aureus* SAMS1, SAMS2, SAMS3, and SA2—Complete gels of Figure 7; Figure S6: Comparison of the affinity of PBP2 for Bocillin™-FL in *S. aureus* SAMS1, SAMS2, SAMS3, and SA2—Complete Gels; Figure S7: Original Images of Figure 3; Table S1: Inhibition zone diameter (mm) obtained in disk diffusion test to *β*-lactam antibiotics; Table S2: Genomes used for global context analysis; Table S3: Genetic changes differing between strains analyzed in this study; Table S4: Mutational frequency of *S. aureus* ATCC 25923, SAMS1 and SA2; Table S5: Number of insertion sequences from *Staphylococcus* sp. per genome detected by ISmapper. Ref. [69] is cited in the Supplementary Materials.
