# Supplementary material for: Genetic and Phenotypic Changes Related to the Development of mec-Independent Oxacillin Non-Susceptibility in ST8 Staphylococcus aureus Recovered after Antibiotic Therapy in a Patient with Bacteremia
Source: Antibiotics (Basel). 2024 Jun 13;13(6):554. doi: 10.3390/antibiotics13060554 (PMC11200602; doi:10.3390/antibiotics13060554)
Supplement: Supplementary file 1 [file antibiotics-13-00554-s001.zip › antibiotics-3008417-supplementary.pdf]

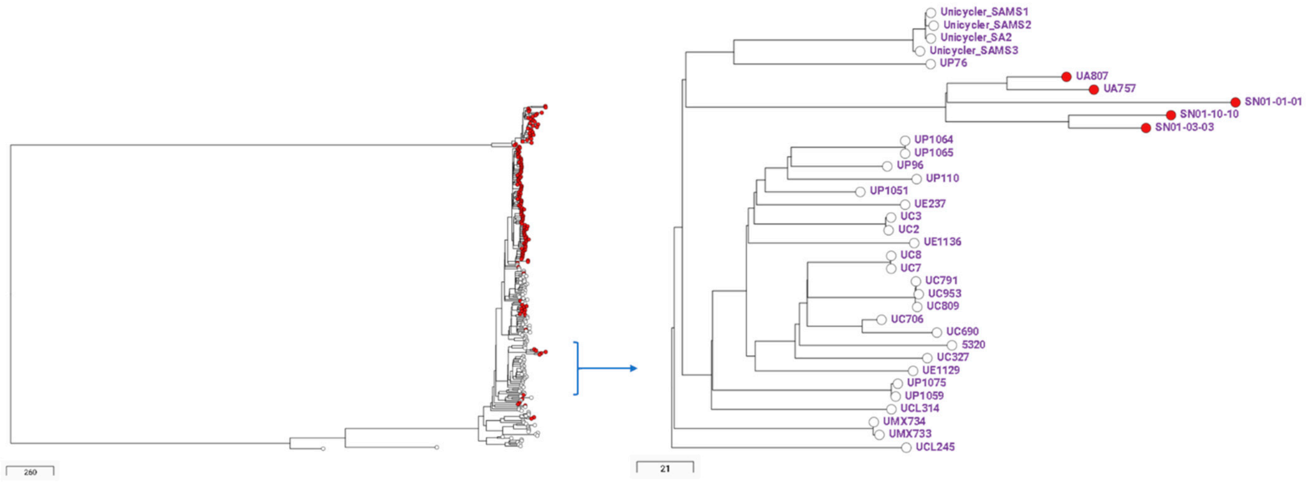

Supplementary Figure S1. CC8 Global context. Phylogenetic tree made in Pathogen Watch with the 4 genomes from this study and 259 CC8 genomes from 19 countries. To the right: Subtree, showing close relationships of strains analyzed in this study. The tree nodes are coloured by the presence of the *mecA* gene. The project is available at: https://pathogen.watch/collection/s4pbk4850a1j-st8-cemic-update-23-06-2023

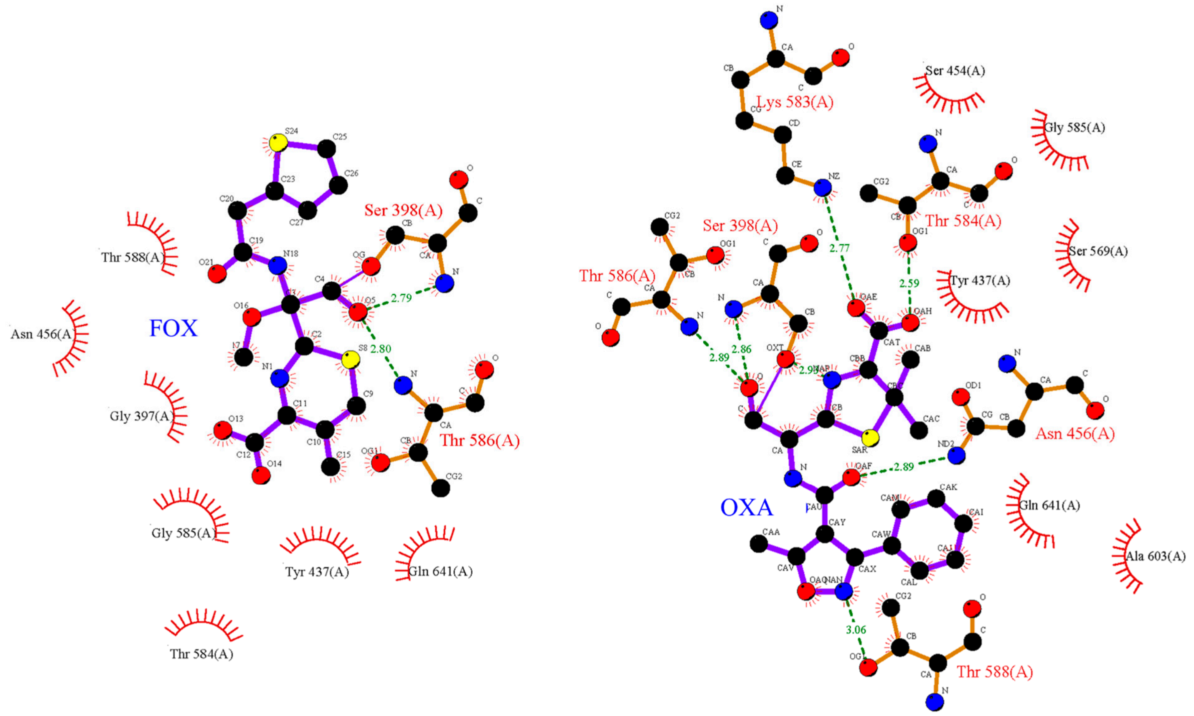

Supplementary Figure S2. 2D view showing the main interactions involved in the complex between SA2-PBP2 and cefoxitin (FOX) or oxacillin (OXA). Ligands are shown in purple. Main hydrogen bonds and hydrophobic interactions are shown as green dotted-lines and red spiked semicircles, respectively. Distances are in angstroms.

A) SAMS1

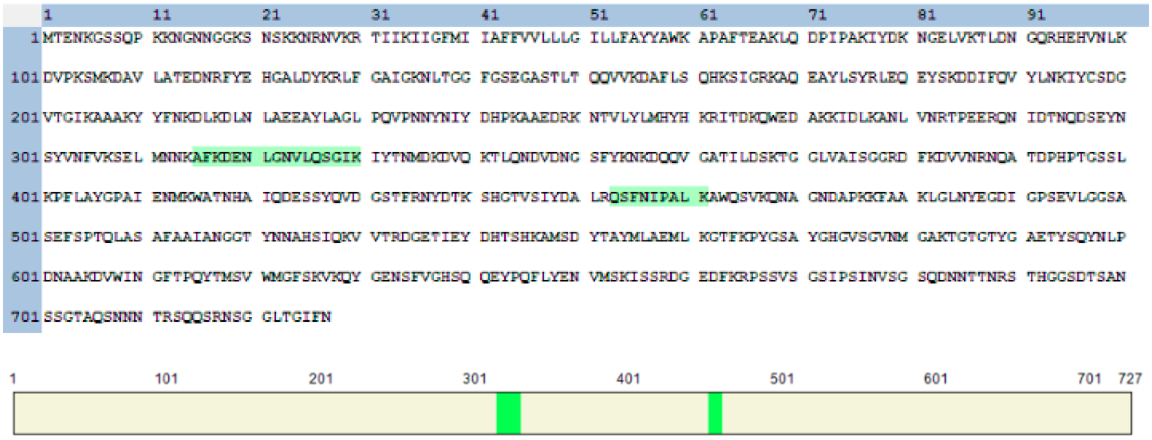

B) SA2

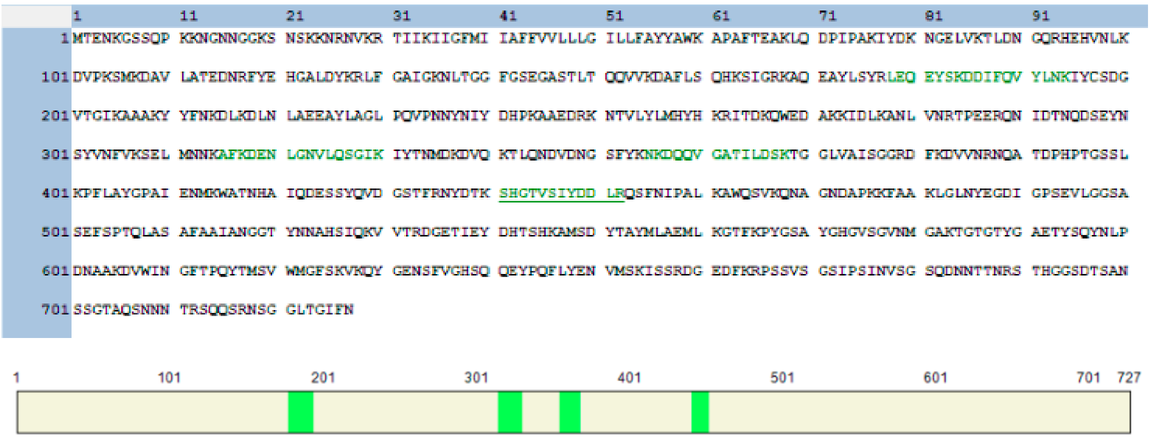

Supplementary Figure S3. LC/MS/MS analysis of PBP2 sequences. Tryptic coverage for PBP2 peptides identified from in-gel digestion of the regions around 70-75 kDa from membrane preparations of *S. aureus* A) SAMS1 and B) SA2. PBP2 was identified in both samples with high confidence (XCorr values > 2.3 at the peptide level). Ref. [69] in main text.

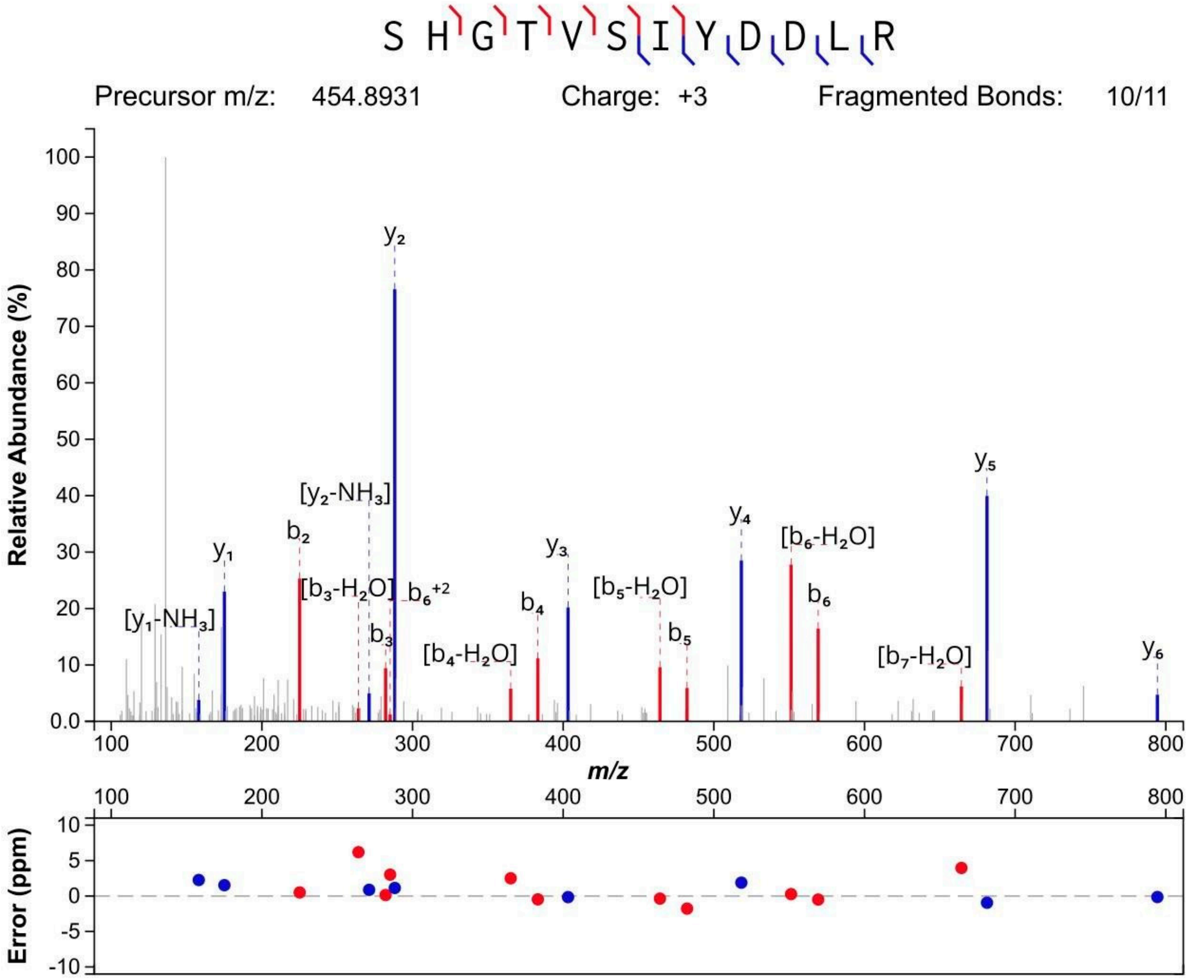

Supplementary Figure S4. Annotated MS/MS spectra of the SHGTVSIYDD450 PBP2 peptide identified in the 70-75 kDa region of membrane protein extract of *S. aureus* SA2.

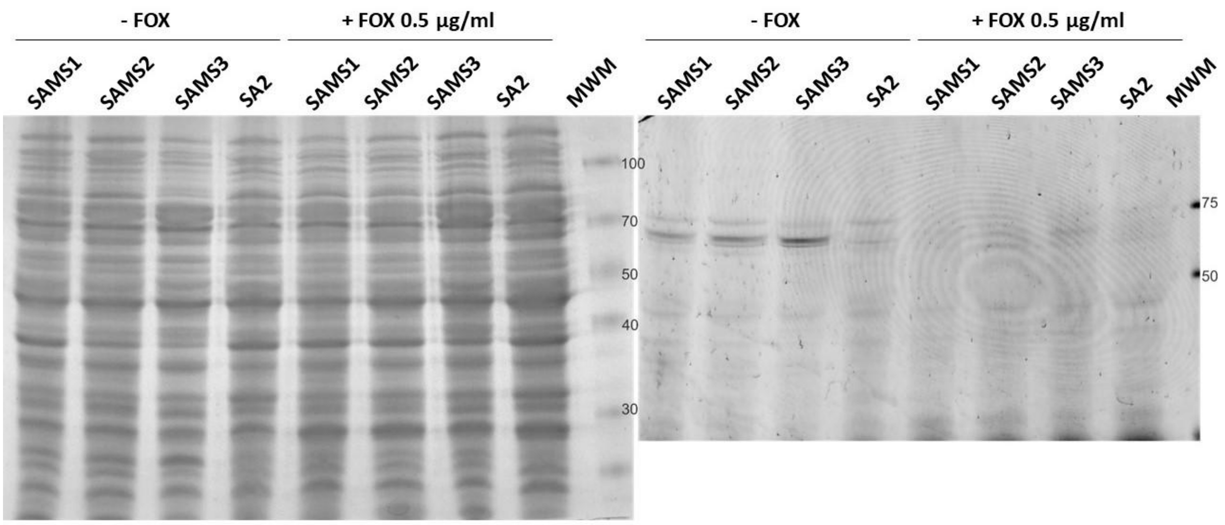

Supplementary Figure S5. Comparison of the affinity of PBP2 for Bocillin™-FL in *S. aureus* SAMS1, SAMS2, SAMS3, and SA2 - COMPLETE GELS of Figure 7. The PBPs were labeled with Bocillin™-FL. Lanes 1-4 show membrane preparations incubated with Bocillin™-FL prior to separation by SDS-PAGE. Lanes 5-8 show membrane preparations from cultures grown with 0.5 µg/ml cefoxitin (FOX), which were then incubated with Bocillin™-FL. Lane 9. Molecular Weight Markers (Blue Plus Protein Marker (Transgen Biotech) + 1/5 dilution of Precision Plus Protein™ WesternC™ Standard (BioRad))**. Left:** 12% SDS-PAGE gel stained with Coomassie Brilliant Blue-stained; 60 µg total protein in each lane; MWM that can be visualized: Blue Plus Protein Marker. **Right:** same gel previously scanned in a Typhoon FLA 7000, FAM filter; Precision Plus Protein™ WesternC™ Standard; bands at 75, 50 and 25 kDa can be visualized with a 532 nm laser or green LED (FAM filter in Typhoon FLA 7000).

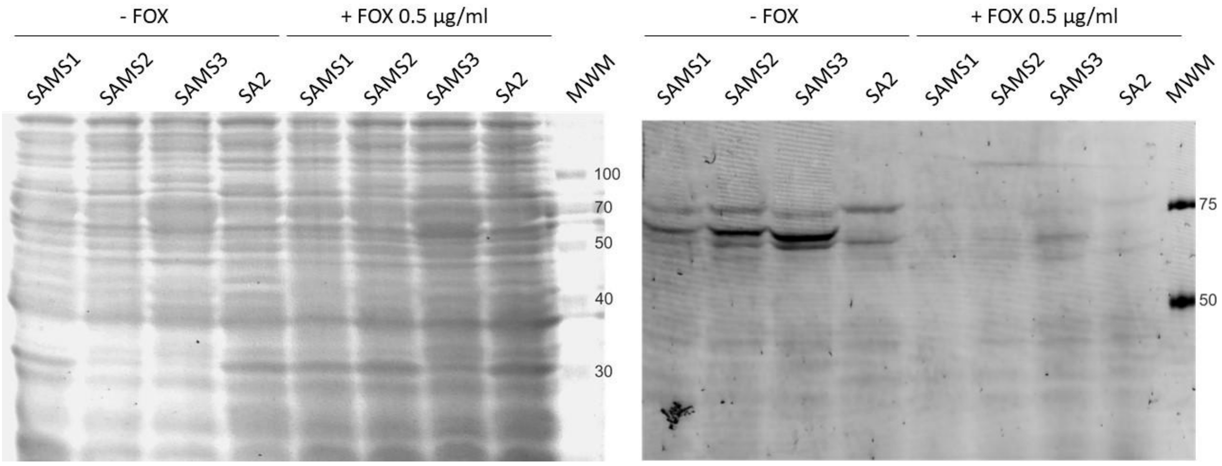

Supplementary Figure S6. Comparison of the affinity of PBP2 for Bocillin™-FL in *S. aureus* SAMS1, SAMS2, SAMS3, and SA2 - COMPLETE GELS. Same samples resolved in Figure S4; 120 µg total protein in each lane. The PBPs were labeled with Bocillin™-FL. Lanes 1-4 show membrane preparations incubated with Bocillin™-FL prior to separation by SDS-PAGE. Lanes 5-8 show membrane preparations from cultures grown with 0.5 µg/ml cefoxitin (FOX), which were then incubated with Bocillin™-FL. Lane 9. Molecular Weight Markers (Blue Plus Protein Marker (Transgen Biotech) + 1/5 dilution of Precision Plus Protein™ WesternC™ Standard (BioRad))**. Left:** 12% SDS-PAGE gel stained with Coomassie Brilliant Blue-stained; 120 µg total protein in each lane; MWM that can be visualized: Blue Plus Protein Marker. **Right:** same gel previously scanned in a Typhoon FLA 7000, FAM filter; Precision Plus Protein™ WesternC™ Standard; bands at 75, 50 and 25 kDa can be visualized with a 532 nm laser or green LED (FAM filter in Typhoon FLA 7000).

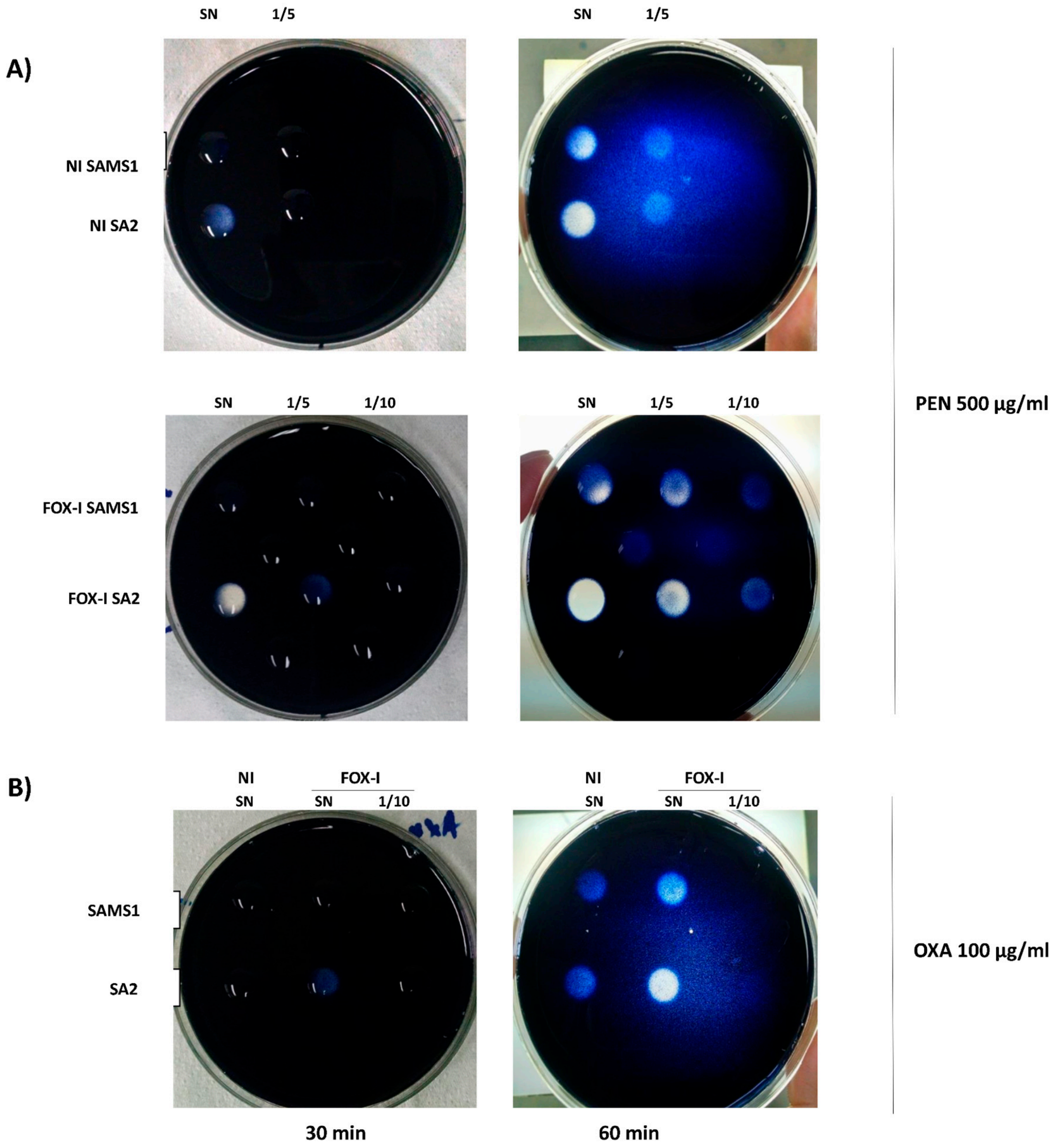

Supplementary Figure S7. Original Images of Figure 3. Comparison of β-lactamase activity by the iodometric method. Serial dilutions of supernatants (1/5, 1/10, 1/20 and 1/50) of non-induced (NI) and FOX-induced (FOX-I) SAMS1 and SA2 cultures were inoculated to starch agar plates containing either penicillin (PEN) 500 µg/ml (A) or oxacillin (OXA) 100 µg/ml (B). The reaction was followed visually during 60 min. SN = Supernatant.

Supplementary Table S1. Inhibition zone diameter (mm) obtained by disk diffusion test to beta-lactam antibiotics

**Antibiotic**	**SAMS1**	**SA2**
Ampicilin (10 µg)	13	8
Ampicilin-sulbactam (10/10 µg)	16	12
Amoxicilin-clavulanic acid (20/10 µg)	20	18
Cephalotin (30 µg)	24	20
Cefotaxime (30 µg)	22	14
Ceftazidime (30 µg)	15	12
Cefepime (30 µg)	20	18
Ceftriaxone (30 µg)	20	18
Imipenem (10 µg)	48	48
Meropenem (10 µg)	32	32
Ertapenem (10 µg)	25	25

Supplementary Table S2. Genomes used for global context analysis

NAME	DATE	STRAIN	COUNTRY	REFERENCE-GROUP	SAMPLE ACCESSION	RUN ASSEMBLY ACCESSION	SOURCE-INFECTION	BIOPROJECT	CITY	CLINICAL ORIGIN	LITERATURE LINK (PMID)	LATITUDE	LONGITUDE
NCTC8325.fasta	1950	NCTC8325	United Kingdom	Reference	SAMN02604235	GCF_000013425.1		PRJNA237				51,7520209	-1,2577263
Staphylococcus_aureus_subsp_aureus_USA300_TCH1516_GCF_000017085_1.fa	2003	USA300_TCH1516	United States of America	Reference	SAMN00253845	GCF_000017085.1		PRJNA19489				37,09024	-95,712891
OC8.fasta	2007	OC8	Rusia	Reference	SAMD00043228	GCA_002355355.1		PRJDB4364				56,0106	92,5201
VC40.fasta	2002	VC40	Germany	Reference	SAMN02603393	GCF_000245495.1		PRJNA224116				51,165691	10.451526000000058
USA500_2395.fasta	1996	USA500_2395	United States of America	Reference	SAMN03290990	GCA_000746505.1		PRJNA240091				37,09024	-95,712891
UMX772	2012		Mexico	Arias, 2017	SAMN03940758	LGWH00000000	Blood	PRJNA291213		Blood	28760895	20,6667	-103,35
UC51	2011		Colombia	Arias, 2017	SAMN03940722	LGWV00000000	Blood	PRJNA291213		Blood	28760895	4,5981	-74,0779887
UC311	2012		Colombia	Arias, 2017	SAMN03940739	LGXM00000000	Blood	PRJNA291213		Blood	28760895	4,5981	-74,0779887
UC312	2012		Colombia	Arias, 2017	SAMN05721742	MIHX00000000	Blood	PRJNA291213		Blood	28760895	4,5981	-74,0779887
UC623	2012		Colombia	Arias, 2017	SAMN03940755	LGWE00000000	Blood	PRJNA291213		Blood	28760895	4,5981	-74,0779887
UCL378	2012		Chile	Arias, 2017	SAMN03940744	LGWM00000000	Blood	PRJNA291213		Blood	28760895	-33,45	-70,6688887

UMX750	2012		Mexico	Arias, 2017	SAMN03940756	LGWF00000000	Blood	PRJNA291213		Blood	28760895	20,6667	-103,35
UV699	2013		Venezuela	Arias, 2017	SAMN05721761	MIHI00000000	Blood	PRJNA291213		Blood	28760895	10,4805937	-66,90361
UMX773	2012		Mexico	Arias, 2017	SAMN05721751	MJHX00000000	Blood	PRJNA291213		Blood	28760895	20,6667	-103,35
UP1073	2013		Peru	Arias, 2017	SAMN05721754	MIHO00000000	Blood	PRJNA291213		Blood	28760895	-12,0433	-77,3084514
UC60	2011		Colombia	Arias, 2017	SAMN03940724	LGWX00000000	Blood	PRJNA291213		Blood	28760895	4,5981	-74,0779887
UC50	2011		Colombia	Arias, 2017	SAMN03940721	LMZC00000000	Blood	PRJNA291213	Bogota	Blood	28760895	4,5981	-74,0779887
UE206	2012		Ecuador	Arias, 2017	SAMN03940734	LGXH00000000	Blood	PRJNA291213		Blood	28760895	-0,2333	-78,5188887
UV185	2012		Venezuela	Arias, 2017	SAMN03940731	LGXE00000000	Blood	PRJNA291213		Blood	28760895	10,4805937	-66,90361
UB576	2012		Brazil	Arias, 2017	SAMN04346842	LPWS00000000	Blood	PRJNA291213	Sao Pablo	Blood	28760895	-23,55	-46,6354887
UC309	2012		Colombia	Arias, 2017	SAMN05721741	MIHY00000000	Blood	PRJNA291213	Bogota	Blood	28760895	4,5981	-74,0779887
UV695	2013		Venezuela	Arias, 2017	SAMN05721760	MIHJ00000000	Blood	PRJNA291213		Blood	28760895	10,4805937	-66,90361
UC622	2012		Colombia	Arias, 2017	SAMN05721743	MIHW00000000	Blood	PRJNA291213	Bogota	Blood	28760895	4,5981	-74,0779887
UE201	2012		Ecuador	Arias, 2017	SAMN03940746	LGWO00000000	Blood	PRJNA291213		Blood	28760895	-0,2333	-78,5188887
UE181	2012		Ecuador	Arias, 2017	SAMN03940730	LGXD00000000	Blood	PRJNA291213		Blood	28760895	-0,2333	-78,5188887
UE232	2012		Ecuador	Arias, 2017	SAMN03940736	LGXJ00000000	Blood	PRJNA291213		Blood	28760895	-0,2333	-78,5188887

UP81	2011		Peru	Arias, 2017	SAMN03940726	LGWZ00000000	Blood	PRJNA291213		Blood	28760895	-12,0433	-77,3084514
UC916	2013		Colombia	Arias, 2017	SAMN03940764	LGXS00000000	Blood	PRJNA291213		Blood	28760895	4,5981	-74,0779887
UV1151	2013		Venezuela	Arias, 2017	SAMN03940774	LGWA00000000	Blood	PRJNA291213		Blood	28760895	10,4805937	-66,90361
UV170	2012		Venezuela	Arias, 2017	SAMN03940729	LGXC00000000	Blood	PRJNA291213		Blood	28760895	10,4805937	-66,90361
UC334	2012		Colombia	Arias, 2017	SAMN03940742	LGXP00000000	Blood	PRJNA291213		Blood	28760895	4,5981	-74,0779887
UC151	2012		Colombia	Arias, 2017	SAMN03940727	LGXA00000000	Blood	PRJNA291213		Blood	28760895	4,5981	-74,0779887
UE203	2012		Ecuador	Arias, 2017	SAMN03940733	LGXG00000000	Blood	PRJNA291213		Blood	28760895	-0,2333	-78,5188887
UA757	2013		Argentina	Arias, 2017	SAMN04346835	LPWL00000000	Blood	PRJNA291213	Buenos Aires	Blood	28760895	-34,603684	-58,38156
UC325	2012		Colombia	Arias, 2017	SAMN03940745	LGWN00000000	Blood	PRJNA291213		Blood	28760895	4,5981	-74,0779887
UC313	2012		Colombia	Arias, 2017	SAMN03940740	LGXN00000000	Blood	PRJNA291213		Blood	28760895	4,5981	-74,0779887
UB563	2012		Brazil	Arias, 2017	SAMN03940750	LGWS00000000	Blood	PRJNA291213	Sao Pablo	Blood	28760895	-23,55	-46,6354887
UP72	2011		Peru	Arias, 2017	SAMN03940725	LGWY00000000	Blood	PRJNA291213		Blood	28760895	-12,0433	-77,3084514
UE220	2012		Ecuador	Arias, 2017	SAMN05721746	MIHT00000000	Blood	PRJNA291213		Blood	28760895	-0,2333	-78,5188887
UC1001	2013		Colombia	Arias, 2017	SAMN03940772	LGVY00000000	Blood	PRJNA291213		Blood	28760895	4,5981	-74,0779887
UMX733	2012		Mexico	Miller, 2018	SAMN13979484	GCA_011025545.1	Blood	PRJNA580194	Guadalajara	Blood	29977970	20,65	-103,34

UE1129	2013		Ecuador	Miller, 2018	SAMN13979383	GCA_011026905.1	Blood	PRJNA580194	Quito	Blood	29977970	-0,18	-78,46
UCL725	2014		Chile	Miller, 2018	SAMN13979353	GCA_011023665.1	Blood	PRJNA580194	Santiago	Blood	29977970	-33,44	-70,66
UC265	2012		Colombia	Miller, 2018	SAMN13979173	GCA_011027855.1	Blood	PRJNA580194	Bogota	Blood	29977970	4,71	-74,07
UC38	2011		Colombia	Miller, 2018	SAMN13979209	GCA_011028645.1	Blood	PRJNA580194	Bogota	Blood	29977970	4,71	-74,07
UB653	2012		Brazil	Miller, 2018	SAMN13979127	GCA_011023085.1	Blood	PRJNA580194	Porto Alegre	Blood	29977970	-30,03	-51,2
UA902	2013		Argentina	Miller, 2018	SAMN13154693	GCA_009674405.1	Blood	PRJNA580194		Blood	29977970	-34,61	-58,37
UC3	2011		Colombia	Miller, 2018	SAMN13979176	GCA_011028745.1	Blood	PRJNA580194	Bogota	Blood	29977970	4,71	-74,07
UCL314	2012		Chile	Miller, 2018	SAMN13979283	GCA_011027805.1	Blood	PRJNA580194	Santiago	Blood	29977970	-33,44	-70,66
UG274	2012		Guatemala	Miller, 2018	SAMN13979447	GCA_011027915.1	Blood	PRJNA580194	Guatemala	Blood	29977970	14,63	-90,5
UCL403	2012		Chile	Miller, 2018	SAMN13979315	GCA_011022715.1	Blood	PRJNA580194	Santiago	Blood	29977970	-33,44	-70,66
UCL343	2012		Chile	Miller, 2018	SAMN13979290	GCA_011023285.1	Blood	PRJNA580194	Santiago	Blood	29977970	-33,44	-70,66
UP1075	2013		Peru	Miller, 2018	SAMN13979513	GCA_011030975.1	Blood	PRJNA580194	Lima	Blood	29977970	-12,04	-77,04
UE193	2012		Ecuador	Miller, 2018	SAMN13979399	GCA_011024835.1	Blood	PRJNA580194	Quito	Blood	29977970	-0,18	-78,46
UA888	2013		Argentina	Miller, 2018	SAMN13154694	GCA_009674995.1	Blood	PRJNA580194		Blood	29977970	-34,61	-58,37
UB422	2012		Brazil	Miller, 2018	SAMN13979062	GCA_011023065.1	Blood	PRJNA580194	Sao Paulo	Blood	29977970	-23,5	-46,6

UC690	2013		Colombia	Miller, 2018	SAMN13979253	GCA_011023785.1	Blood	PRJNA580194	Bogota	Blood	29977970	4,71	-74,07
UC2	2011		Colombia	Miller, 2018	SAMN13979165	GCA_011025225.1	Blood	PRJNA580194	Bogota	Blood	29977970	4,71	-74,07
UE216	2012		Ecuador	Miller, 2018	SAMN13979414	GCA_011027795.1	Blood	PRJNA580194	Quito	Blood	29977970	-0,18	-78,46
UG281	2012		Guatemala	Miller, 2018	SAMN13979449	GCA_011027985.1	Blood	PRJNA580194	Guatemala	Blood	29977970	14,63	-90,5
UCL404	2012		Chile	Miller, 2018	SAMN13979316	GCA_011023165.1	Blood	PRJNA580194	Santiago	Blood	29977970	-33,44	-70,66
UP1065	2013		Peru	Miller, 2018	SAMN13979509	GCA_011025865.1	Blood	PRJNA580194	Lima	Blood	29977970	-12,04	-77,04
UC809	2013		Colombia	Miller, 2018	SAMN13979273	GCA_011024245.1	Blood	PRJNA580194	Bogota	Blood	29977970	4,71	-74,07
UA705	2013		Argentina	Miller, 2018	SAMN13154684	GCA_009674275.1	Blood	PRJNA580194		Blood	29977970	-34,61	-58,37
UC8	2011		Colombia	Miller, 2018	SAMN13979272	GCA_011025425.1	Blood	PRJNA580194	Bogota	Blood	29977970	4,71	-74,07
UCL391	2012		Chile	Miller, 2018	SAMN13979312	GCA_011022465.1	Blood	PRJNA580194	Santiago	Blood	29977970	-33,44	-70,66
UA905	2013		Argentina	Miller, 2018	SAMN13154691	GCA_009675205.1	Blood	PRJNA580194		Blood	29977970	-34,61	-58,37
UA751	2013		Argentina	Miller, 2018	SAMN13154685	GCA_009675825.1	Blood	PRJNA580194		Blood	29977970	-34,61	-58,37
UP1012	2013		Peru	Miller, 2018	SAMN13979495	GCA_011023525.1	Blood	PRJNA580194	Lima	Blood	29977970	-12,04	-77,04
UA868	2013		Argentina	Miller, 2018	SAMN13154688	GCA_009676165.1	Blood	PRJNA580194		Blood	29977970	-34,61	-58,37
UP1051	2013		Peru	Miller, 2018	SAMN13979504	GCA_011023925.1	Blood	PRJNA580194	Lima	Blood	29977970	-12,04	-77,04

UCL393	2012		Chile	Miller, 2018	SAMN13979313	GCA_011022795.1	Blood	PRJNA580194	Santiago	Blood	29977970	-33,44	-70,66
UC33	2011		Colombia	Miller, 2018	SAMN13979198	GCA_011027065.1	Blood	PRJNA580194	Bogota	Blood	29977970	4,71	-74,07
UB522	2012		Brazil	Miller, 2018	SAMN13979081	GCA_011022595.1	Blood	PRJNA580194	Sao Paulo	Blood	29977970	-23,5	-46,6
UE192	2012		Ecuador	Miller, 2018	SAMN13979398	GCA_011024505.1	Blood	PRJNA580194	Quito	Blood	29977970	-0,18	-78,46
UA830	2013		Argentina	Miller, 2018	SAMN13154681	GCA_009675725.1	Blood	PRJNA580194		Blood	29977970	-34,61	-58,37
UB498	2012		Brazil	Miller, 2018	SAMN13979072	GCA_011034335.1	Blood	PRJNA580194	Sao Paulo	Blood	29977970	-23,5	-46,6
UV158	2012		Venezuela	Miller, 2018	SAMN13979562	GCA_011025045.1	Blood	PRJNA580194	Caracas	Blood	29977970	10,4683841	-66,9604067
UC327	2012		Colombia	Miller, 2018	SAMN13979195	GCA_011022745.1	Blood	PRJNA580194	Bogota	Blood	29977970	4,71	-74,07
UA844	2013		Argentina	Miller, 2018	SAMN13154687	GCA_009675925.1	Blood	PRJNA580194		Blood	29977970	-34,61	-58,37
UCL763	2012		Chile	Miller, 2018	SAMN13979357	GCA_011024575.1	Blood	PRJNA580194	Concepcion	Blood	29977970	-36,82	-73,04
UB520	2012		Brazil	Miller, 2018	SAMN13979079	GCA_011023405.1	Blood	PRJNA580194	Sao Paulo	Blood	29977970	-23,5	-46,6
UA924	2013		Argentina	Miller, 2018	SAMN13154677	GCA_009676145.1	Blood	PRJNA580194		Blood	29977970	-34,61	-58,37
UP76	2011		Peru	Miller, 2018	SAMN13979540	GCA_011025935.1	Blood	PRJNA580194	Lima	Blood	29977970	-12,04	-77,04
UA832	2013		Argentina	Miller, 2018	SAMN13154682	GCA_009675475.1	Blood	PRJNA580194		Blood	29977970	-34,61	-58,37
UA843	2013		Argentina	Miller, 2018	SAMN13154686	GCA_009674325.1	Blood	PRJNA580194		Blood	29977970	-34,61	-58,37

UG989	2012		Guatemala	Miller, 2018	SAMN13979479	GCA_011026415.1	Blood	PRJNA580194	Guatemala	Blood	29977970	14,63	-90,5
UA806	2013		Argentina	Miller, 2018	SAMN13154689	GCA_009674305.1	Blood	PRJNA580194		Blood	29977970	-34,61	-58,37
UC5	2011		Colombia	Miller, 2018	SAMN13979225	GCA_011030715.1	Blood	PRJNA580194	Bogota	Blood	29977970	4,71	-74,07
UC791	2013		Colombia	Miller, 2018	SAMN13979271	GCA_011024085.1	Blood	PRJNA580194	Bogota	Blood	29977970	4,71	-74,07
UC706	2012		Colombia	Miller, 2018	SAMN13979260	GCA_011030925.1	Blood	PRJNA580194	Bogota	Blood	29977970	4,71	-74,07
UB521	2012		Brazil	Miller, 2018	SAMN13979080	GCA_011022565.1	Blood	PRJNA580194	Sao Paulo	Blood	29977970	-23,5	-46,6
UP96	2011		Peru	Miller, 2018	SAMN13979550	GCA_011024735.1	Blood	PRJNA580194	Lima	Blood	29977970	-12,04	-77,04
UE1136	2013		Ecuador	Miller, 2018	SAMN13979386	GCA_011023845.1	Blood	PRJNA580194	Quito	Blood	29977970	-0,18	-78,46
UG1091	2013		Guatemala	Miller, 2018	SAMN13979431	GCA_011028365.1	Blood	PRJNA580194	Guatemala	Blood	29977970	14,63	-90,5
UB390	2012		Brazil	Miller, 2018	SAMN13979061	GCA_011022475.1	Blood	PRJNA580194	Sao Paulo	Blood	29977970	-23,5	-46,6
UE237	2012		Ecuador	Miller, 2018	SAMN13979426	GCA_011031965.1	Blood	PRJNA580194	Quito	Blood	29977970	-0,18	-78,46
UCL245	2012		Chile	Miller, 2018	SAMN13979281	GCA_011027575.1	Blood	PRJNA580194	Santiago	Blood	29977970	-33,44	-70,66
UCL708	2014		Chile	Miller, 2018	SAMN13979343	GCA_011027895.1	Blood	PRJNA580194	Santiago	Blood	29977970	-33,44	-70,66
UC61	2011		Colombia	Miller, 2018	SAMN13979238	GCA_011031305.1	Blood	PRJNA580194	Bogota	Blood	29977970	4,71	-74,07
UV1141	2013		Venezuela	Miller, 2018	SAMN13979554	GCA_011024895.1	Blood	PRJNA580194	Caracas	Blood	29977970	10,4683841	-66,9604067

UP86	2011		Peru	Miller, 2018	SAMN13979545	GCA_011023685.1	Blood	PRJNA580194	Lima	Blood	29977970	-12,04	-77,04
UC7	2011		Colombia	Miller, 2018	SAMN13979257	GCA_011026285.1	Blood	PRJNA580194	Bogota	Blood	29977970	4,71	-74,07
UA903	2013		Argentina	Miller, 2018	SAMN13154690	GCA_009675265.1	Blood	PRJNA580194		Blood	29977970	-34,61	-58,37
UP114	2011		Peru	Miller, 2018	SAMN13979523	GCA_011023865.1	Blood	PRJNA580194	Lima	Blood	29977970	-12,04	-77,04
UG276	2012		Guatemala	Miller, 2018	SAMN13979448	GCA_011027785.1	Blood	PRJNA580194	Guatemala	Blood	29977970	14,63	-90,5
UP116	2011		Peru	Miller, 2018	SAMN13979525	GCA_011026345.1	Blood	PRJNA580194	Lima	Blood	29977970	-12,04	-77,04
UC62	2011		Colombia	Miller, 2018	SAMN13979239	GCA_011025645.1	Blood	PRJNA580194	Bogota	Blood	29977970	4,71	-74,07
UA920	2013		Argentina	Miller, 2018	SAMN13154692	GCA_009676085.1	Blood	PRJNA580194		Blood	29977970	-34,61	-58,37
UP1064	2013		Peru	Miller, 2018	SAMN13979508	GCA_011025185.1	Blood	PRJNA580194	Lima	Blood	29977970	-12,04	-77,04
UCL722	2014		Chile	Miller, 2018	SAMN13979352	GCA_011027505.1	Blood	PRJNA580194	Santiago	Blood	29977970	-33,44	-70,66
UP110	2011		Peru	Miller, 2018	SAMN13979521	GCA_011024555.1	Blood	PRJNA580194	Lima	Blood	29977970	-12,04	-77,04
UA831	2013		Argentina	Miller, 2018	SAMN13154683	GCA_009676015.1	Blood	PRJNA580194		Blood	29977970	-34,61	-58,37
UC953	2013		Colombia	Miller, 2018	SAMN13979276	GCA_011031485.1	Blood	PRJNA580194	Bogota	Blood	29977970	4,71	-74,07
UP1059	2013		Peru	Miller, 2018	SAMN13979506	GCA_011026035.1	Blood	PRJNA580194	Lima	Blood	29977970	-12,04	-77,04
UMX734	2012		Mexico	Miller, 2018	SAMN13979485	GCA_011025255.1	Blood	PRJNA580194	Guadalajara	Blood	29977970	20,65	-103,34

UC146	2012		Colombia	Miller, 2018	SAMN13979155	GCA_011026525.1	Blood	PRJNA580194	Bogota	Blood	29977970	4,71	-74,07
UG957	2012		Guatemala	Miller, 2018	SAMN13979465	GCA_011024785.1	Blood	PRJNA580194	Guatemala	Blood	29977970	14,63	-90,5
UE194	2012		Ecuador	Miller, 2018	SAMN13979400	GCA_011024885.1	Blood	PRJNA580194	Quito	Blood	29977970	-0,18	-78,46
UE195	2012		Ecuador	Miller, 2018	SAMN13979401	GCA_011027045.1	Blood	PRJNA580194	Quito	Blood	29977970	-0,18	-78,46
UC34	2011		Colombia	Miller, 2018	SAMN13979205	GCA_011027135.1	Blood	PRJNA580194	Bogota	Blood	29977970	4,71	-74,07
5351	2018		Colombia	Carvajal, 2020	SAMN13979045	GCA_010571035.1	Blood	PRJNA595347	Pereira	Blood	32071048	4,81428	-75,69456
5320	2018		Colombia	Carvajal, 2020	SAMN13979042	GCA_010571055.1	Blood	PRJNA595347	Pereira	Blood	32071048	4,81428	-75,69456
5155	2018		Colombia	Carvajal, 2020	SAMN13979025	GCA_010569415.1	Blood	PRJNA595347	Pereira	Blood	32071048	4,81428	-75,69456
UE228			Ecuador	Castro, 2020	SAMN14171719	GCA_011063885.1	Blood	PRJNA595928		Blood	32562543	-0,21	-78,5
UC63			Colombia	Castro, 2020	SAMN14171690	GCA_011062255.1	Blood	PRJNA595928		Blood	32562543	4,6	-74,08
UC693			Colombia	Castro, 2020	SAMN14171694	GCA_011391975.1	Blood	PRJNA595928		Blood	32562543	4,6	-74,08
UA856			Argentina	Castro, 2020	SAMN14171581	GCA_011062115.1	Blood	PRJNA595928	Buenos Aires	Blood	32562543	-34,6158037	-58,5033388
UC17			Colombia	Castro, 2020	SAMN14171684	GCA_011062225.1	Blood	PRJNA595928		Blood	32562543	4,6	-74,08
UC283			Colombia	Castro, 2020	SAMN14171686	GCA_011063485.1	Blood	PRJNA595928		Blood	32562543	4,6	-74,08
UMX784			Mexico	Castro, 2020	SAMN14171754	GCA_011058975.1	Blood	PRJNA595928		Blood	32562543	20,65	-103,34

UC28			Colombia	Castro, 2020	SAMN14171685	GCA_011062325.1	Blood	PRJNA595928		Blood	32562543	4,6	-74,08
UC692			Colombia	Castro, 2020	SAMN14171693	GCA_011063725.1	Blood	PRJNA595928		Blood	32562543	4,6	-74,08
UC150			Colombia	Castro, 2020	SAMN14171682	GCA_011059085.1	Blood	PRJNA595928		Blood	32562543	4,6	-74,08
UE218			Ecuador	Castro, 2020	SAMN14171717	GCA_011063735.1	Blood	PRJNA595928		Blood	32562543	-0,21	-78,5
UE227			Ecuador	Castro, 2020	SAMN14171718	GCA_011063695.1	Blood	PRJNA595928		Blood	32562543	-0,21	-78,5
UE1110			Ecuador	Castro, 2020	SAMN14171710	GCA_011777425.1	Blood	PRJNA595928		Blood	32562543	-0,21	-78,5
UE1105			Ecuador	Castro, 2020	SAMN14171709	GCA_011060285.1	Blood	PRJNA595928		Blood	32562543	-0,21	-78,5
UV1176			Venezuela	Castro, 2020	SAMN14171784	GCA_011059915.1	Blood	PRJNA595928		Blood	32562543	10,48	-66,9
UC554			Colombia	Castro, 2020	SAMN14171688	GCA_011059455.1	Blood	PRJNA595928		Blood	32562543	4,6	-74,08
UE1119			Ecuador	Castro, 2020	SAMN14171712	GCA_011060905.1	Blood	PRJNA595928		Blood	32562543	-0,21	-78,5
UC113			Colombia	Castro, 2020	SAMN14171680	GCA_011061785.1	Blood	PRJNA595928		Blood	32562543	4,6	-74,08
UV1170			Venezuela	Castro, 2020	SAMN14171781	GCA_011391995.1	Blood	PRJNA595928		Blood	32562543	10,48	-66,9
UC155			Colombia	Castro, 2020	SAMN14171683	GCA_011061065.1	Blood	PRJNA595928		Blood	32562543	4,6	-74,08
UG975			Guatemala	Castro, 2020	SAMN14171742	GCA_011061975.1	Blood	PRJNA595928		Blood	32562543	14,63	-90,5
UV1178			Venezuela	Castro, 2020	SAMN14171785	GCA_011058965.1	Blood	PRJNA595928		Blood	32562543	10,48	-66,9

UV1173			Venezuela	Castro, 2020	SAMN14171782	GCA_011059405.1	Blood	PRJNA595928		Blood	32562543	10,48	-66,9
UP1168			Peru	Castro, 2020	SAMN14171770	GCA_011061615.1	Blood	PRJNA595928		Blood	32562543	-12,04	-77,02
UE1097	2013		Ecuador	Castro, 2020	SAMN13577550	GCA_013367295.1	Blood	PRJNA595928		Blood	32562543	-0,21	-78,5
UA807			Argentina	Castro, 2020	SAMN14171573	GCA_011059355.1	Blood	PRJNA595928	Buenos Aires	Blood	32562543	-34,6158037	-58,5033388
UE1135			Ecuador	Castro, 2020	SAMN14171716	GCA_011059685.1	Blood	PRJNA595928		Blood	32562543	-0,21	-78,5
UC691			Colombia	Castro, 2020	SAMN14171692	GCA_011060965.1	Blood	PRJNA595928		Blood	32562543	4,6	-74,08
UE1116			Ecuador	Castro, 2020	SAMN14171711	GCA_011060725.1	Blood	PRJNA595928		Blood	32562543	-0,21	-78,5
UV1174			Venezuela	Castro, 2020	SAMN14171783	GCA_011062395.1	Blood	PRJNA595928		Blood	32562543	10,48	-66,9
UC121			Colombia	Castro, 2020	SAMN14171681	GCA_011061845.1	Blood	PRJNA595928		Blood	32562543	4,6	-74,08
UE1130			Ecuador	Castro, 2020	SAMN14171715	GCA_011392195.1	Blood	PRJNA595928		Blood	32562543	-0,21	-78,5
BRA36	1997		Brazil	Harris, 2010	SAMEA1029559	ERS049878	NA	PRJEB2489	Sao Pablo		20093474	-23,5852	-46,7232
URU110	1998		Uruguay	Harris, 2010	SAMEA1029540	ERS049872	NA	PRJEB2489	Montevideo		20093474	-34,8669	-56,1667
RA6	1996		Argentina	Castillo Ramirez, 2012	SAMEA862591	ERS010982	SSSI	PRJEB2173			23270620	-34	-64
CHL1	1997		Chile	Harris, 2010	SAMEA1029526	ERS049876	NA	PRJEB2489	Santiago		20093474	-33,45	-70,6667
CHL151	1998		Chile	Harris, 2010	SAMEA1029539	ERS049877	NA	PRJEB2489	Santiago		20093474	-33,45	-70,6667

RA3	1996		Argentina	Castillo Ramirez, 2012	SAMEA862588	ERS010981	NA	PRJEB2173			23270620	-34	-64
AGT1	1997		Argentina	Harris, 2010	SAMEA1029527	ERS049887	Urine	PRJEB2489	Buenos Aires		20093474	-34,5997	-58,3819
AGT120	1998		Argentina	Harris, 2010	SAMEA1029554	ERS049880	SSSI	PRJEB2489	Buenos Aires		20093474	-34,5997	-58,3819
URU34	1997		Uruguay	Harris, 2010	SAMEA1029544	ERS049886	NA	PRJEB2489	Montevideo		20093474	-34,8669	-56,1667
RA7	1996		Argentina	Castillo Ramirez, 2012	SAMEA862616	ERS010999	Wound	PRJEB2173			23270620	-34	-64
BRA2	1997		Brazil	Harris, 2010	SAMEA1029514	ERS049875	NA	PRJEB2489	Sao Pablo		20093474	-23,5852	-46,7232
BZ48	1997		Brazil	Harris, 2010	SAMEA1029508	ERS049879	NA	PRJEB2489	Sao Pablo		20093474	-23,5852	-46,7232
AGT9	1997		Argentina	Harris, 2010	SAMEA1029509	ERS049884	Respiratory	PRJEB2489	Buenos Aires		20093474	-34,5997	-58,3819
2028P	2005		Argentina	Herrera, 2021	SAMN13091954	SRS5588740	SSSI	PRJNA577848	ParanÃ¡		33588015	-31,73	-60,52
STV101L	12th June 2012		Colombia	Nurjadi, 2019	SAMN09862508	GCA_003571275.1	abscess	PRJNA486096			30315958	4,35	-74,4
STV563L	21st October 2015		Cuba	Nurjadi, 2019	SAMN09862510	GCA_003571265.1	abscess	PRJNA486096			30315958	23,11	-82,36
STV600L	3rd March 2016		Cuba	Nurjadi, 2019	SAMN09862512	GCA_003571175.1	abscess	PRJNA486096			30315958	23,11	-82,36
STV108L	20th June 2012		Suriname	Nurjadi, 2019	SAMN09862503	GCA_003571365.1	abscess	PRJNA486096			30315958	5,51	-55,1

STV232L	24th June 2013		Cuba	Nurjadi, 2019	SAMN09862504	GCA_003571195.1	abscess	PRJNA486096			30315958	23,11	-82,36
STV658L	23rd September 2016		Cuba	Nurjadi, 2019	SAMN09862511	GCA_003571225.1	abscess	PRJNA486096			30315958	23,11	-82,36
STV209L	10th May 2013		Colombia	Nurjadi, 2019	SAMN09862509	GCA_003571245.1	abscess	PRJNA486096			30315958	4,35	-74,4
HUV5	2006		Colombia	Planet, 2015	SAMN02767311	GCA_001045995.2	Blood	PRJNA246401		Blood	26048971	3,2514	-76,312
M109	2006		Colombia		SAMN02777080	GCA_001045975	Nasal swab - healthy carrier	PRJNA247403	Putumayo			1,15	-76,61667
917			Brazil		SAMN03160716	GCA_000816715.1	Blood	PRJNA265867		Blood		-11	-53
HC1340	2001		Brazil	Botelho, 2019	SAMN03835492	GCA_001515745.1	Nasal swab - healthy carrier	PRJNA288685	Rio de Janeiro		30873127	-22,911	-43,2094
Gv51	1997		Brazil	Botelho, 2019	SAMN03835466	GCA_001515665.1	Bronchial lavage	PRJNA288680	Teresina		30873127	-5,0896	-42,8096
Be62	1996		Brazil	Botelho, 2019	SAMN03765155	GCA_001515685.1	Blood	PRJNA286121	Belem	Blood	30873127	-1,4506	-48,4682
Gv69	29th June 1996		Brazil	Botelho, 2016	SAMN03144721	GCA_000769575.1	Wound	PRJNA264181	Teresina		27152133	-5,0896	-42,8096
CA12	2007		Colombia	Planet, 2015	SAMN02767090	GCA_001045795.2	Blood	PRJNA246373		Blood	26048971	10,24	-75,3
C329	2006		Colombia	Castro, 2020	SAMN02777076	GCA_001045935.1	Blood	PRJNA247399	Medellin	Blood	32562543	6,2308	-75,5906
M121	2004		Colombia	Planet, 2015	SAMN02746692	GCA_001021875.1	Nasal swab - healthy carrier	PRJNA246311			26048971	3,81667	-73,91667

Gv88	1998		Brazil	Botelho, 2019	SAMN03835488	GCA_001515705.1	Wound	PRJNA288682	Teresina		30873127	-5,0896	-42,8096
CA15	2007		Colombia	Planet, 2015	SAMN02767179	GCA_001021895.1	Blood	PRJNA246386		Blood	26048971	10,24	-75,3
V2200	13th September 2007		Venezuela	Planet, 2015	SAMN02743812	GCA_001046095.2	Osteomyelitis	PRJNA246185			26048971	10,5	-66,9167
HC1335	2001		Brazil	Botelho, 2016	SAMN03835494	GCA_001515765.1	Nasal swab - healthy carrier	PRJNA288686	Rio de Janeiro		27635055	-22,911	-43,2094
Bmb9393	1993		Brazil	Cerqueira Costa, 2013	SAMN02603524	GCA_000418345.1	Blood	PRJNA196937	Rio de Janeiro	Blood	23929475	-22,911	-43,2094
SN02-06-01	17th April 2019	NA	Bolivia	Di Gregorio, 2023	SAMEA7470446	ERR8575139	Blood	PRJEB37318		Blood	37227244	-17,38655073	-66,1480427
SN02-03-03	4th September 2019	NA	Bolivia	Di Gregorio, 2023	SAMEA7470416	ERR8575091	Blood	PRJEB37318		Blood	37227244	-19,58281842	-65,7657364
SN02-10-01	28th April 2019	NA	Bolivia	Di Gregorio, 2023	SAMEA7470467	ERR8575195	Blood	PRJEB37318		Blood	37227244	-19,00822038	-65,30747618
SN02-05-10	23rd August 2019	NA	Bolivia	Di Gregorio, 2023	SAMEA7470443	ERR8575138	Blood	PRJEB37318		Blood	37227244	-17,39364519	-66,20557491
SN04-02-08	21st April 2019	NA	Paraguay	Di Gregorio, 2023	SAMEA7470375	ERR8575034	Blood	PRJEB37318		Blood	37227244	-25,2712998	-57,5861739
SN01-19-04	28th August 2019	NA	Argentina	Di Gregorio, 2023	SAMEA7470209	ERR8575105	Blood	PRJEB37318		Blood	37227244	-32,8721203	-68,8659946
SN01-03-03	17th August 2019	NA	Argentina	Di Gregorio, 2023	SAMEA7470011	ERR8575137	Blood	PRJEB37318		Blood	37227244	-41,132588	-71,320732

SN05-06-08	27th May 2019	NA	Uruguay	Di Gregorio, 2023	SAMEA8748036	ERR9884537	Blood	PRJEB37318		Blood	37227244	-34,8950484	-56,1941862
SN01-02-02	13th June 2019	NA	Argentina	Di Gregorio, 2023	SAMEA7469993	ERR8575112	Blood	PRJEB37318		Blood	37227244	-41,13616	-71,300119
SN01-29-05	10th May 2019	NA	Argentina	Di Gregorio, 2023	SAMEA7470349	ERR8575282	Blood	PRJEB37318		Blood	37227244	-31,3652037	-58,0154752
SN02-09-04	14th May 2019	NA	Bolivia	Di Gregorio, 2023	SAMEA7470460	ERR8575176	Blood	PRJEB37318		Blood	37227244	-19,04477046	-65,26290938
SN02-14-01	17th April 2019	NA	Bolivia	Di Gregorio, 2023	SAMEA7470482	ERR8575221	Blood	PRJEB37318		Blood	37227244	-17,76754107	-63,15771873
SN01-20-06	1st July 2019	NA	Argentina	Di Gregorio, 2023	SAMEA7470222	ERR8575121	Blood	PRJEB37318		Blood	37227244	-31,7406031	-60,5254518
SN04-03-05	25th June 2019	NA	Paraguay	Di Gregorio, 2023	SAMEA7470388	ERR8575053	Blood	PRJEB37318		Blood	37227244	-25,3576798	-57,5461316
SN02-16-03	26th September 2019	NA	Bolivia	Di Gregorio, 2023	SAMEA8747950	ERR9884459	Blood	PRJEB37318		Blood	37227244	-14,82175136	-64,89597954
SN01-10-04	2nd May 2019	NA	Argentina	Di Gregorio, 2023	SAMEA7470110	ERR8575236	Blood	PRJEB37318		Blood	37227244	-34,5922517	-58,4038097
SN01-08-08	18th May 2019	NA	Argentina	Di Gregorio, 2023	SAMEA7470098	ERR8575286	Blood	PRJEB37318		Blood	37227244	-34,5987057	-58,3999683
SN01-12-03	5th May 2019	NA	Argentina	Di Gregorio, 2023	SAMEA7470145	ERR8575284	Blood	PRJEB37318		Blood	37227244	-34,4778621	-58,9113611
SN01-02-01	14th May 2019	NA	Argentina	Di Gregorio, 2023	SAMEA7469991	ERR8575109	Blood	PRJEB37318		Blood	37227244	-41,13616	-71,300119
SN01-02-07	3rd September 2019	NA	Argentina	Di Gregorio, 2023	SAMEA7470001	ERR8575122	Blood	PRJEB37318		Blood	37227244	-41,13616	-71,300119

SN01-12-01	25th April 2019	NA	Argentina	Di Gregorio, 2023	SAMEA7470141	ERR8575279	Blood	PRJEB37318		Blood	37227244	-34,4778621	-58,9113611
SN01-11-06	13th May 2019	NA	Argentina	Di Gregorio, 2023	SAMEA7470131	ERR8575264	Blood	PRJEB37318		Blood	37227244	-34,5970481	-58,4240376
SN01-10-09	17th August 2019	NA	Argentina	Di Gregorio, 2023	SAMEA7470117	ERR8575252	Blood	PRJEB37318		Blood	37227244	-34,5922517	-58,4038097
SN01-28-02	20th April 2019	NA	Argentina	Di Gregorio, 2023	SAMEA7470332	ERR8575254	Blood	PRJEB37318		Blood	37227244	-53,7819919	-67,7011113
SN01-08-05	6th May 2019	NA	Argentina	Di Gregorio, 2023	SAMEA7470092	ERR8575216	Blood	PRJEB37318		Blood	37227244	-34,5987057	-58,3999683
SN01-19-01	22nd April 2019	NA	Argentina	Di Gregorio, 2023	SAMEA7470206	ERR8575099	Blood	PRJEB37318		Blood	37227244	-32,8721203	-68,8659946
SN05-05-06	10th June 2019	NA	Uruguay	Di Gregorio, 2023	SAMEA8748027	ERR9884529	Blood	PRJEB37318		Blood	37227244	-34,9080058	-56,2141358
SN05-05-07	28th July 2019	NA	Uruguay	Di Gregorio, 2023	SAMEA8748028	ERR9884530	Blood	PRJEB37318		Blood	37227244	-34,9080058	-56,2141358
SN01-08-02	7th June 2019	NA	Argentina	Di Gregorio, 2023	SAMEA7470086	ERR8575207	Blood	PRJEB37318		Blood	37227244	-34,5987057	-58,3999683
SN05-01-05	16th October 2019	NA	Uruguay	Di Gregorio, 2023	SAMEA8747997	ERR9884509	Blood	PRJEB37318		Blood	37227244	-34,3670681	-57,8200244
SN01-26-01	16th April 2019	NA	Argentina	Di Gregorio, 2023	SAMEA7470304	ERR8575062	Blood	PRJEB37318		Blood	37227244	-27,4712228	-58,8344856
SN01-10-10	18th September 2019	NA	Argentina	Di Gregorio, 2023	SAMEA7470119	ERR8575255	Blood	PRJEB37318		Blood	37227244	-34,5922517	-58,4038097

SN01-12-02	4th May 2019	NA	Argentina	Di Gregorio, 2023	SAMEA7470143	ERR8575281	Blood	PRJEB37318		Blood	37227244	-34,4778621	-58,9113611
SN02-10-05	26th October 2019	NA	Bolivia	Di Gregorio, 2023	SAMEA7470470	ERR8575202	Blood	PRJEB37318		Blood	37227244	-19,00822038	-65,30747618
SN02-03-06	22nd September 2019	NA	Bolivia	Di Gregorio, 2023	SAMEA7470423	ERR8575101	Blood	PRJEB37318		Blood	37227244	-19,58281842	-65,7657364
SN01-26-09	9th July 2019	NA	Argentina	Di Gregorio, 2023	SAMEA7470320	ERR8575234	Blood	PRJEB37318		Blood	37227244	-27,4712228	-58,8344856
SN01-24-04	2nd June 2019	NA	Argentina	Di Gregorio, 2023	SAMEA7470291	ERR8575193	Blood	PRJEB37318		Blood	37227244	-32,9512946	-60,6751806
SN01-27-06	19th April 2019	NA	Argentina	Di Gregorio, 2023	SAMEA7470333	ERR8575244	Blood	PRJEB37318		Blood	37227244	-43,2493016	-65,3098291
SN02-09-10	11th September 2019	NA	Bolivia	Di Gregorio, 2023	SAMEA7470465	ERR8575192	Blood	PRJEB37318		Blood	37227244	-19,04477046	-65,26290938
SN01-12-07	3rd October 2019	NA	Argentina	Di Gregorio, 2023	SAMEA7470152	ERR8575298	Blood	PRJEB37318		Blood	37227244	-34,4778621	-58,9113611
SN02-10-08	28th September 2019	NA	Bolivia	Di Gregorio, 2023	SAMEA7470478	ERR8575211	Blood	PRJEB37318		Blood	37227244	-19,00822038	-65,30747618
SN01-13-10	22nd May 2019	NA	Argentina	Di Gregorio, 2023	SAMEA7470167	ERR8575032	Blood	PRJEB37318		Blood	37227244	-34,6487158	-58,5174063
SN02-09-02	15th April 2019	NA	Bolivia	Di Gregorio, 2023	SAMEA7470456	ERR8575170	Blood	PRJEB37318		Blood	37227244	-19,04477046	-65,26290938
SN01-04-01	7th August 2019	NA	Argentina	Di Gregorio, 2023	SAMEA7470017	ERR8575144	Blood	PRJEB37318		Blood	37227244	-27,382825	-55,890695

SN01-11-07	13th May 2019	NA	Argentina	Di Gregorio, 2023	SAMEA7470133	ERR8575267	Blood	PRJEB37318		Blood	37227244	-34,5970481	-58,4240376
SN01-14-03	3rd July 2019	NA	Argentina	Di Gregorio, 2023	SAMEA7470172	ERR8575039	Blood	PRJEB37318		Blood	37227244	-38,7201224	-62,2578026
SN03-07-05	15th July 2019	NA	Brasil	Di Gregorio, 2023	SAMEA8747985	ERR9884500	Blood	PRJEB37318		Blood	37227244	-27,5980058	-48,5191903
SN01-01-05	4th June 2019	NA	Argentina	Di Gregorio, 2023	SAMEA7469981	ERR8575093	Blood	PRJEB37318		Blood	37227244	-41,138239	-71,312615
SN01-10-05	8th May 2019	NA	Argentina	Di Gregorio, 2023	SAMEA7470112	ERR8575240	Blood	PRJEB37318		Blood	37227244	-34,5922517	-58,4038097
SN01-08-07	20th July 2019	NA	Argentina	Di Gregorio, 2023	SAMEA7470095	ERR8575220	Blood	PRJEB37318		Blood	37227244	-34,5987057	-58,3999683
SN03-07-10	21st March 2019	NA	Brasil	Di Gregorio, 2023	SAMEA8747993	ERR9884505	Blood	PRJEB37318		Blood	37227244	-27,5980058	-48,5191903
SN01-04-02	26th June 2019	NA	Argentina	Di Gregorio, 2023	SAMEA7470019	ERR8575147	Blood	PRJEB37318		Blood	37227244	-27,382825	-55,890695
SN02-07-02	1st October 2019	NA	Bolivia	Di Gregorio, 2023	SAMEA7470453	ERR8575164	Blood	PRJEB37318		Blood	37227244	-17,97573561	-67,11377773
SN01-08-03	1st May 2019	NA	Argentina	Di Gregorio, 2023	SAMEA7470089	ERR8575210	Blood	PRJEB37318		Blood	37227244	-34,5987057	-58,3999683
SN01-29-02	22nd April 2019	NA	Argentina	Di Gregorio, 2023	SAMEA7470343	ERR8575272	Blood	PRJEB37318		Blood	37227244	-31,3652037	-58,0154752
SN01-01-01	25th April 2019	NA	Argentina	Di Gregorio, 2023	SAMEA7469973	ERR8575081	Blood	PRJEB37318		Blood	37227244	-41,138239	-71,312615
SN04-04-06	19th April 2019	NA	Paraguay	Di Gregorio, 2023	SAMEA7470404	ERR8575075	Blood	PRJEB37318		Blood	37227244	-25,4479985	-57,3643143

SN02-14-02	20th April 2019	NA	Bolivia	Di Gregorio, 2023	SAMEA7470484	ERR8575078	Blood	PRJEB37318		Blood	37227244	-17,76754107	-63,15771873
SN03-07-02	21st May 2019	NA	Brasil	Di Gregorio, 2023	SAMEA8747982	ERR9884497	Blood	PRJEB37318		Blood	37227244	-27,5980058	-48,5191903
SN01-13-09	21st May 2019	NA	Argentina	Di Gregorio, 2023	SAMEA7470165	ERR8575029	Blood	PRJEB37318		Blood	37227244	-34,6487158	-58,5174063
SN03-02-05	15th July 2019	NA	Brasil	Di Gregorio, 2023	SAMEA8747961	ERR9884473	Blood	PRJEB37318		Blood	37227244	-22,9012241	-43,2909239
SN01-26-06	8th June 2019	NA	Argentina	Di Gregorio, 2023	SAMEA7470317	ERR8575226	Blood	PRJEB37318		Blood	37227244	-27,4712228	-58,8344856
SN01-16-09	20th August 2019	NA	Argentina	Di Gregorio, 2023	SAMEA7470197	ERR8575067	Blood	PRJEB37318		Blood	37227244	-24,772612	-65,417795
SN01-28-04	27th June 2019	NA	Argentina	Di Gregorio, 2023	SAMEA7470336	ERR8575260	Blood	PRJEB37318		Blood	37227244	-53,7819919	-67,7011113
CD16-145	2016	CD16-145	Brazil	Viana, 2021	SAMN16946788	GCA_021008415.1	Nasal Swab	PRJNA378176		Nasal Swab	34670645	-22,911	-43,2094
CD15-176	2015	CD15-176	Brazil	Viana, 2021	SAMN16946703	GCA_021010155.1	Blood	PRJNA378176		Blood	34670645	-22,911	-43,2094
CD16-121	2016	CD16-121	Brazil	Viana, 2021	SAMN16946780	GCA_021008595.1	NBIS	PRJNA378176		NBIS	34670645	-22,911	-43,2094
Unicycler_SAMS2	19th September 2013	SAMS2	Argentina	This study			Blood		Buenos Aires	Blood	This study	-34,5572367	-58,4953249
Unicycler_SA2	16th November 2013	SA2	Argentina	This study			Blood		Buenos Aires	Blood	This study	-34,5572367	-58,4953249
Unicycler_SAMS3	2nd November 2013	SAMS3	Argentina	This study			Blood		Buenos Aires	Blood	This study	-34,5572367	-58,4953249

Unicycler_SAMS 1	5th August 2013	SAMS1	Argentina	This study			Blood		Buenos Aires	Blood	This study	-34,5572367	-58,4953249

Supplementary Table S3. Genetic changes differing between strains analyzed in this study after mapping the reads to the *S. aureus* SAMS1 genome. All changes and positions are expressed in reference to the *S. aureus* USA300 FPR3757 reference genome except otherwise stated. Genes without an annotated name are in reference to the CDS of USA300 by their locus tag.

SAMS1 Contig Number	Position	Type	SAMS1 (ref)	SAMS2	SAMS3	SA2	LOCUS_TAG	GENE	PRODUCT	EFFECT	Nucleotide Position	Amino Acid Position
1	242850	snp	A	G	A	A	ABOBFFPD_00233	yjbH	hypothetical protein	synonymous_variant	c.756T>C	p.Tyr252Tyr
1	468489	del	AT	A	A	A	ABOBFFPD_00448	stp1	protein phosphatase 2C domain-containing protein	frameshift_variant	c.19delT	p.Tyr7fs
1	468517	ins	C	C	CT	C	ABOBFFPD_00448	stp1	protein phosphatase 2C domain-containing protein	frameshift_variant	c.44dupT	p.Leu15fs
2	170450	snp	T	G	T	T	ABOBFFPD_00790	adhE	bifunctional acetaldehyde-CoA/alcohol dehydrogenase	missense_variant	c.2480A>C	p.Glu827Ala
3	335008	snp	T	G	G	G	ABOBFFPD_01354	aroB	putative 3-dehydroquinate synthase	missense_variant	c.991T>G	p.Tyr331Asp
3	351502	snp	G	G	G	T	ABOBFFPD_01369	pbp2	glycosyl transferase family protein	missense_variant	c.1349C>A	p.Ala450Asp
6	24600	snp	G	T	G	G	ABOBFFPD_01932	yvqF	hypothetical protein	missense_variant	c.521C>A	p.Pro174Gln
6	24667	snp	C	C	C	T	ABOBFFPD_01932	yvqF	hypothetical protein	missense_variant	c.454G>A	p.Ala152Thr
6	100955	snp	C	C	C	T	ABOBFFPD_02003	SAUSA300_RS10620	phage head-tail joining protein	missense_variant	c.247G>A	p.Glu83Lys
8	81057	snp	C	C	T	C	ABOBFFPD_02310	cls2	phospholipase D/Transphosphatidylase	missense_variant	c.683G>A	p.Arg228His
8	83621	snp	T	C	C	C	ABOBFFPD_02314	rodA	FtsW/RodA/SpoVE family cell division protein	missense_variant	c.530A>G	p.Glu177Gly
8	44717	ins	G	G	G	GA	ABOBFFPD_02271	rpoE	DNA-directed RNA polymerase subunit delta	frameshift_variant	c.42dupA	p.Ser15fs

Supplementary Table S4. Mutational frequency of *S. aureus* ATCC 25923, SAMS1 and SA2 expressed as the number of RIF-resistant mutants recovered compared to the total viable count of each culture. The average of 3 independent experiments is shown.

**Strain**	**Mutational Frequency**
S. aureus ATCC25923	< 1.3 x 10 -7
SAMS1	7 x 10 -7
SA2	9.3 x 10 -7

Supplementary Table S5. Number of insertion sequences from Staphylococcus sp. per genome detected by ISmapper

	**SAMS1**	**SAMS2**	**SAMS3**	**SA2**
IS1181	4	3	5	4
IS1182	0	0	0	0
IS1272	14	6	9	14
IS256	0	0	0	0
IS431mec	0	0	0	0
ISSau2	2	1	4	4
ISSau3	10	9	11	11
ISSau4	0	0	0	0
ISSau5	0	0	0	0
ISSau6	0	0	0	0
ISSau8	4	3	5	2
ISSau9	0	0	0	0
ISSep1	11	3	8	3
ISSep2	1	0	3	1
ISSep3	3	2	3	3
ISSha1	0	0	0	0
ISsau1	0	0	0	0
